# Low-threshold anisotropic polychromatic emission from monodisperse quantum dots

**DOI:** 10.1093/nsr/nwae311

**Published:** 2024-09-05

**Authors:** Yangzhi Tan, Wai Yuen Fu, Hemin Lin, Dan Wu, Xiao Wei Sun, Hoi Wai Choi, Kai Wang

**Affiliations:** Institute of Nanoscience and Applications, Department of Electrical and Electronic Engineering, Southern University of Science and Technology, Shenzhen 518055, China; Department of Electrical and Electronic Engineering, The University of Hong Kong, Hong Kong 999077, China; Department of Electrical and Electronic Engineering, The University of Hong Kong, Hong Kong 999077, China; Institute of Nanoscience and Applications, Department of Electrical and Electronic Engineering, Southern University of Science and Technology, Shenzhen 518055, China; College of New Materials and New Energies, Shenzhen Technology University, Shenzhen 518118, China; Institute of Nanoscience and Applications, Department of Electrical and Electronic Engineering, Southern University of Science and Technology, Shenzhen 518055, China; Department of Electrical and Electronic Engineering, The University of Hong Kong, Hong Kong 999077, China; Institute of Nanoscience and Applications, Department of Electrical and Electronic Engineering, Southern University of Science and Technology, Shenzhen 518055, China

**Keywords:** colloidal quantum dots, multi-excitonic emission, microcavity

## Abstract

Colloidal quantum dots (QDs) are solution-processable semiconductor nanocrystals with favorable optoelectronic characteristics, one of which is their multi-excitonic behavior that enables broadband polychromatic light generation and amplification from monodisperse QDs. However, the practicality of this has been limited by the difficulty in achieving spatial separation and patterning of different colors as well as the high pumping intensity required to excite the multi-excitonic states. Here, we have addressed these issues by integrating monodisperse QDs in multi-excitonic states into a specially designed cavity, in which the QDs exhibit an anisotropic polychromatic emission (APE) characteristic that allows for tuning the emission from green to red by shifting the observation direction from perpendicular to lateral. Subsequently, the APE threshold under 300-ps pulsed excitation has been reduced from 32 to 21 μJ cm^−2^ by optimizing the cavity structure. Based on the manipulation of multi-excitonic emission and angle-dependent wavelength selectivity of the developed cavity, we have fabricated a full-color micro-pixel array with a pixel size as small as 23 μm by combining cavity-integrated monodisperse QDs and blue backlight. Furthermore, the threshold of APE under quasi-continuous-wave pumping was as low as 5 W cm^−2^, indicating its compatibility with commercial LEDs and/or laser diodes. Since APE arises from the multi-excitonic behavior of QDs that supports optical gain, its unprecedentedly low threshold suggests the feasibility of the diode-pumped colloidal QD laser. This work demonstrates a novel method of manipulating the QDs’ optical properties beyond controlling their size, composition or structure, and reveals great potential for achieving full-color emission using monodisperse QDs.

## INTRODUCTION

Colloidal quantum dots (QDs) are zero-dimensional semiconductor nanocrystals whose optoelectronic properties can be tuned over a wide range by adjusting their composition, size and structure, making them promising for diverse applications such as display [1], photovoltaics [2] and sensing [3]. Specifically, the 3D quantum confinement in colloidal QDs results in their atomic-like discrete electronic states, whose wide inter-band separation inhibits the thermal depopulation of band-edge states and thereby reduces the optical gain and lasing threshold and improves the thermal stability of lasing characteristics [4,5]. These properties make colloidal QDs an attractive gain medium for realizing solution-processable laser diodes compatible with easily scalable and low-cost fabrication and integration techniques.

The realization of optical gain in colloidal QDs requires population inversion that occurs in their multi-excitonic states [6]. For CdSe-based QDs with 2-fold degeneracy, the theoretical gain threshold corresponds to an average per-dot excitonic occupancy <*N*> of 1 [7]. Interestingly, when CdSe-based QDs are stimulated to excitonic states with <*N*> exceeding 5 through intense optical and/or electrical excitation, several high-energy transition paths, including the 1S_e_–1S_lh_ (referred to as 1S’) and 1P_e_–1P_hh_ (1P) transitions, in addition to the band-edge 1S_e_–1S_hh_ transition (1S), can be observed [5,8,9]. For example, Klimov *et al.* have demonstrated multi-band photoluminescence (PL) and electroluminescence (EL) from monodisperse CdSe-based continuously graded (cg) QDs [10], in which the cg-QDs exhibit an EL peak at 1.96 eV (*λ* = 633 nm) which is corresponding to the band-edge 1S transition when the injected current density *J* is below 41 A cm^−2^. As the *J* gradually increases to 1 kA cm^−2^, two additional peaks indicating the 1S’ (2.02 eV, *λ* = 614 nm) and 1P (2.1 eV, *λ* = 590 nm) transitions appear successively. Notably, the full width at half maximum (FWHM) of the 1P transition is ∼150 meV or 42 nm. Those findings show that the light generation and amplification range of the monodisperse CdSe QDs could be expanded to cover the red, yellow and green regions concurrently, indicating the potential to achieve full-color emission by coupling the blue backlights with monodisperse QDs. However, although there have been many reports on the multi-excitonic polychromatic emission from monodisperse QDs [9–22], their applicability is still limited owing to several problems. First, previously reported multi-excitonic PL and EL from CdSe QDs are pumped by fs-pulse laser and/or intense current injection of several A cm^−2^ [10,14–16,18–20,22–24]. For practical application, the threshold of multi-excitonic emission should be further reduced to a level that can be achieved by portable laser diodes (LDs) and/or micro-LEDs operating in continuous wave (cw) or quasi-cw modes. Second, different colors should be separated from the broad multi-band emission spectrum spatially and/or temporally. This separation should address two fundamental challenges: spectral narrowing and pixelation. For applications like displays and communications, where narrow emission linewidths are essential, color separation is necessary to isolate specific spectral regions. In display applications, the realization of full-color patterns requires the precise arrangement of different color pixels. Spatial separation enables the definition of these pixels by assigning specific wavelengths to distinct spatial locations, leading to the formation of full-color images. In essence, color separation unlocks the potential of QD multi-excitonic emission for practical applications by providing a means to achieve both narrow-band emission and full-color pixelation. The last challenge is to define the polychromatic subpixels with monodisperse QDs in a high resolution, and at large scale and low cost.

Here, we have addressed these issues and shown a patterned red and green micro-pixel array. A pixel size below 50 μm can be achieved using the monodisperse CdZnSe/ZnSe/ZnCdS QDs with an intrinsic spontaneous emission (1S transition) peak of 631 nm and FWHM of 23 nm. First, under ps-pulse laser excitation, three-band PL with peaks at 568 nm (1P), 595 nm (1S’) and 631 nm (1S) from QDs was observed above a threshold of 55 μJ cm^−2^. Next, the QDs were integrated into an angle-dependent wavelength-selective cavity constructed by Ag thin film and 16-pair Ta_2_O_5_/SiO_2_ distributed Bragg reflectors (DBRs) to separately extract the green and red emission from the three-band PL to the in-plane and out-of-plane directions, respectively. Such a unique phenomenon is called anisotropic polychromatic emission (APE), in which the emission color is shifted from green to red suddenly when the emission angle (*θ*, defined as the angle between out-of-plane z-axis and observation direction) exceeds 45°. To further improve the efficiency of APE, a LiF spacer layer was introduced between the QDs and Ag to suppress the surface plasmon absorption, enhance the constructive Purcell effect and improve the light extraction. Consequently, the intensity of green and red emission is relatively increased by ∼2.5 and 2.1 times, respectively. Also, the APE threshold (*P_th_,_APE_*) has been decreased from 32 to 21 μJ cm^−2^ under ps-pulse excitation. Then, by patterning the Ag thin film with a fine metal mask (FMM), we have fabricated a red and green micro-pixel array with a pixel size as small as 23 μm using the monodisperse QDs, by leveraging the manipulation of the multi-excitonic emission characteristic and the angle-dependent wavelength selectivity facilitated by the developed cavity. When combined with blue backlights, a full-color micro-pixel array has been achieved. It is worth mentioning that this is the first work that simultaneously achieves polychromatic emission, separation of different colors and full-color pixelation using monodisperse colloidal QDs. More importantly, we demonstrated that the APE can be stimulated by quasi-cw pump laser above a threshold as low as ∼5 W cm^−2^, indicating that this monodisperse QD-based color converting scheme is compatible with commercially available LEDs and/or LDs. Notably, the APE is essentially based on multi-excitonic behavior of the CdSe QDs that potentially supports optical gain, and the threshold for triggering it is unprecedentedly low thanks to the coupled cavity with positive Purcell effect. It is of great interest to explore the feasibility of diode-pumped colloidal QD lasers.

## RESULTS

### Pulse laser excited multi-band PL

**Figure 1.**
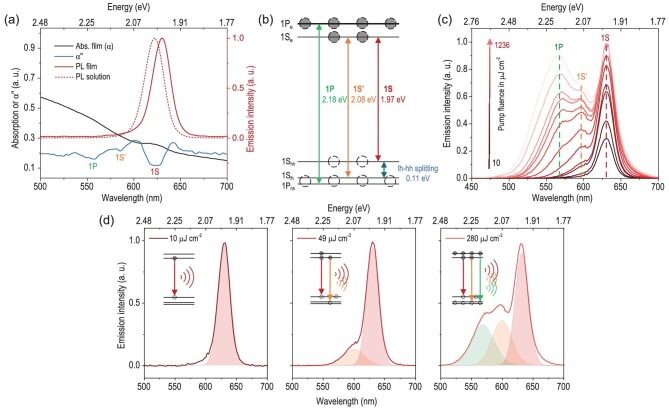
Optical properties of the monodisperse CdZnSe/ZnSe/ZnCdS QDs. (a) The photoluminescent spontaneous emission spectra of the QD solution (red dashed line) and film (red solid line), absorption spectrum (*α*, black line) and its second derivative (d^2^*α*/d*λ*^2^, marked as *α*’’, blue line). (b) The derived band-edge states (1P_e_, 1S_e_, 1S_hh_, 1S_lh_ and 1P_hh_) and three transition paths (1S, 1S’ and 1P) of QDs. (c) The emission spectra of QDs under variable pumping fluence from 10 μJ cm^−2^ to 1236 μJ cm^−2^. (d) The emission spectra and corresponding peak fitting of QDs under pumping fluence of 10 μJ cm^−2^ (left), 49 μJ cm^−2^ (middle) and 280 μJ cm^−2^ (right).

The QDs used in this study have a type-I CdZnSe/ZnSe/ZnCdS core/shell/shell structure and an average diameter of 12.6 nm, as characterized by a transmission electron microscope (TEM) (see Fig. S1). The analysis of their size distribution shows a standard deviation of 1.5 nm, demonstrating a great uniformity enabled by high quality synthesis. As shown in Fig. 1a, the monodisperse QDs with uniform morphology also exhibit narrow-band spontaneous emission peaking at 622 nm (or 1.99 eV) and 631 nm (1.97 eV) with a FWHM of 24 nm (0.077 eV) and 23 nm (0.072 eV) in solution and film, respectively, corresponding to the band-edge 1S transition. The photoluminescence quantum yield (PLQY) of the QD solution and film is 96% and 80%, respectively. From the linear absorption (*α*) spectrum of the QD film and its second-order derivative (d^2^*α*/d*λ*^2^, marked as *α*′′), three paths of 1S, 1S′ and 1P transitions can be well distinguished. The light-heavy hole splitting (Δ_lh-hh_) is ∼110 meV, twice that (56 meV) reported in the CdSe cg-QDs [14,25], indicating a strong asymmetric compression of the CdZnSe core by engineered graded shell structure and suppressed thermal depopulation, which are helpful to reduce the optical gain threshold [26]. The energy of the 1P transition is ∼2.18 eV, 218 meV larger than that (1.97 eV) of the 1S transition. The near-band-edge states and three transition paths mentioned above, derived from the PL and *α*’’ spectra, are illustrated in Fig. 1b.

To demonstrate the multi-excitonic radiative recombination, we excited the QDs via a 355 nm pulse laser with a pulse duration of 300 ps, repetition rate of 100 Hz and adjustable pumping fluence. The PL characterization set-up is illustrated in Fig. S2. Figure 1c shows the PL spectra of the QD film under different pump fluences, in which the 1S, 1S′ and 1P transitions successively emerge when increasing the fluence from 10 μJ cm^−2^ to 1236 μJ cm^−2^, corresponding to the gradual transition from single- to two- and three-band PL. As illustrated in Fig. 1d (left), the QDs exhibit single-band PL at 631 nm under a pumping fluence of 10 μJ cm^−2^, indicating an absolute 1S transition with <*N*> smaller than 1. When the pumping fluence is increased to 49 μJ cm^−2^, or accordingly, the <*N*> is above 2, a 1S’ transition band at 597 nm (2.08 eV) can be extracted from the PL spectrum by peak fitting, as shown in the middle of Fig. 1d. When the fluence is increased to 280 μJ cm^−2^ to achieve a <*N*> more than 6, an extra 1P transition at 568 nm (2.18 eV) emerges, indicating the complete filling of the 2-fold-degenerate 1S_e_ band, which supports saturated band-edge optical gain [9,14]. The fitting results coincide well with those shown in Fig. 1b, derived from the data in Fig. 1a. The thresholds of the 1S′ and 1P transitions are ∼22 μJ cm^−2^ and 55 μJ cm^−2^, respectively. Furthermore, a two-band random lasing action with a threshold of ∼26 μJ cm^−2^ has been demonstrated, as illustrated in Fig. S3, when focusing the pumping light on the edge of the QD film on quartz substrate, providing proof of the strong optical gain from QDs under pulse excitation.

### APE from cavity-coupled QDs

**Figure 2.**
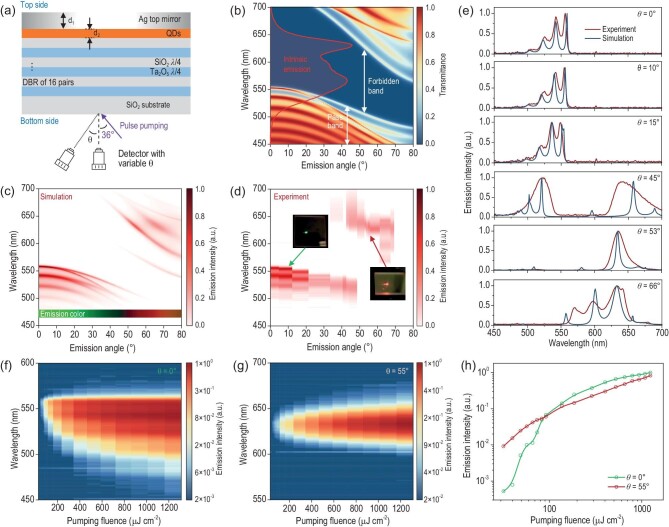
APE characteristics of the cavity-coupled QDs. (a) The structure of the cavity consists of an Ag thin film, sandwiched QD layer and the bottom 16-pair Ta_2_O_5_/SiO_2_ DBR. The pumping light is incident to the bottom side of the sample with an oblique of ∼36° to improve the effective absorption of the excitation by QDs. (b) DBR transmittance spectra with various *θ* from 0° to 80° and the intrinsic emission spectrum of QDs under pumping fluence of 1236 μJ cm^−2^. (c) Simulated emission spectra of the cavity-coupled QDs with different *θ* from 0° to 80°. (d) Characterized emission spectra of the sample with different *θ* from 0° to 68° and (e) a comparison between the simulation results. (f) Emission spectra of the cavity-coupled QDs under pumping fluence *P* from 32 to 1236 μJ cm^−2^ at *θ* = 0°. (g) Emission spectra of the cavity-coupled QDs under pumping fluence *P* from 32 to 1236 μJ cm^−2^ at *θ* = 55°. (h) Pumping fluence dependent relative emission intensity of the cavity-coupled QDs at *θ* = 0° and 55°.

Separation of distinct emission colors in the broad multi-excitonic emission band is essential for practical application. It enables narrow-band emission for displays and communications, and precise pixel definition for full-color displays, unlocking the potential practicality of the multi-excitonic emission of QDs. To extract the red and green emissions from broad-band emitting QDs, they are sandwiched into the cavity constructed by a top Ag thin film and 16-pair Ta_2_O_5_/SiO_2_ DBR deposited on a 1 mm thick SiO_2_ substrate (see Fig. 2a) that supports strong angle-dependent wavelength selectivity. The thickness of the QDs (*d_2_*) is ∼62 nm, as characterized by the step profiler. The Ag thickness (*d_1_*) is optimized to 50 nm to support an average reflectance of ∼95% between 500 nm and 700 nm and relatively efficient light extraction, which is discussed in the later section. The optimization process of the thickness of Ag and QDs can be found in Fig. S4. The DBR is designed to exhibit a maximum reflectance of normal incidence larger than 99.9% at 635 nm and fabricated by plasma-assisted e-beam evaporation. The refractive indices of each material and the characterization of DBR are given in Fig. S5. The entire fabrication process is illustrated in Fig. S6. The pulse laser is incident obliquely on the bottom side of the sample at an angle of 36° relative to the normal direction.

The angle-dependent wavelength selectivity is mainly attributed to the dispersion characteristic of the DBR, which is essentially a 1D photonic crystal structure whose photonic forbidden band can be tuned by changing the wave vector, or more directly, the incident/emission angle *θ* [27]. Figure 2b shows the transmittance dispersion of the DBR used here, where the forbidden band undergoes a blue-shift and narrows as *θ* increases. In contrast, the passband from 450 nm to 550 nm also experiences a blue-shift when *θ* is increased from 0° to 30°, while another passband covering the red wavelengths emerges when *θ* is further increased to 80°. The overlap region between the intrinsic emission spectrum of the QD film under a pumping fluence of 1236 μJ cm^−2^ and the variable passband gradually changes from green to red.

As a result, the emission color from the cavity-coupled QDs should also change with the same tendency. Figure 2c shows its simulated emission spectra at different *θ*, in which the emission is initially green and the peaks blue-shift slightly with increased *θ* when it is below 45°. When *θ* reaches 45°, the red emission achieves a balance with the green one. The emission is suddenly changed from green to red if the *θ* is further increased. The change of the corresponding Commission Internationale de l’Eclairage (CIE) coordinates in [X, Y] with various *θ* from 0° to 80° can be found in Fig. S7, showing a great adjustable range from [0.144, 0.678] to [0.639, 0.325]. The test result (see Fig. 2d) matches well with the simulation, as the observed emission color changes from green to red (see the insets of Fig. 2d) at different viewing angles. Although it is difficult to obtain data above 68° due to the limit of our characterization platform, the APE phenomenon has been well demonstrated in practice. Figure 2e compares the simulation and characterization results at certain angles, confirming the overall accuracy of the simulation modeling, especially at small angles. Larger discrepancies between experimental and simulated results at larger angles likely stem from our PL characterization set-up. The 50 × objective with numerical aperture of 0.45 collects light over a large spot size of ∼200 μm and a wide angle (26.7°). Therefore, the collected spectrum should be an integral over a range of angles, rather than the signal of a specific emission angle. More accurate results could hopefully be obtained by back focal plane (BFP) imaging [28] for example.

The pumping fluence dependent APE characteristic has also been investigated. Figure 2f and g show the emission spectra of the cavity-coupled QDs under pumping fluence *P* from 32 to 1236 μJ cm^−2^, emitting at *θ* = 0° and 55°, respectively. When the *P* is smaller than 32 μJ cm^−2^, the green emission at *θ* = 0° is unobservable or too weak to be detected. An emission peak at 559 nm can be clearly distinguished in Fig. 2f when *P* = 32 μJ cm^−2^, indicating the action of APE. Notably, the *P_th_,_APE_* of ∼32 μJ cm^−2^ is even smaller than that (55 μJ cm^−2^) of the 1P transition, which could be attributed to the positive Purcell effect that describes the enhancement of the spontaneous emission rate in a cavity compared to that in free space, which shall be discussed in detail in the later section. The spectrum is gradually broadened with increased *P* because of the stepwise filling of the 1P_e_ band when the <*N*> is larger than 5. A similar phenomenon can also be observed in the emission spectrum of QD film, as shown in Fig. 1c. In contrast, the emission peak at *θ* = 55° maintains at ∼634 nm, and only the relative intensity has been changed with various *P*. Figure 2h compares the *P*-dependent emission intensity of the cavity-coupled QDs emitting at *θ* = 0° and 55°. For the green emission at *θ* = 0°, the intensity exhibits a non-linear enhancement with increased *P*. In contrast, the red emission intensity at *θ* = 55° is almost proportional to *P*. As a result, although the green emission is much weaker than the red one at low pump fluence just above the *P_th_,_APE_*, their intensity is nearly of the same order when the *P* is larger than 50 μJ cm^−2^.

Moreover, the sample was pumped under a *P* of 1236 μJ cm^−2^ for several hours and subsequently placed in atmosphere with constant temperature of 22°C and relative humidity of ∼70% for 155 days without any encapsulation, to investigate its long-term stability, which is essential for practical application. Figure S8 depicts the variation of green emission intensity at 0° for the control sample, without a LiF spacer layer, with continuous operation for 100 hours under a *P* of 1236 μJ cm⁻². The results demonstrate a PL intensity decay below 5%, indicating the great operational stability of the APE sample. Furthermore, after 24 hours of operation and 155 days of storage in atmosphere without encapsulation, the degraded sample exhibits a similar *P_th_,_APE_* to the fresh one: ∼32 μJ cm^−2^ (see Fig. S9). Specifically, at low pump fluence, below 60 μJ cm^−2^, the emission intensity of the degraded sample at 0° is even higher than that of the fresh one. Still, when pumping fluence exceeds 200 μJ cm^−2^, the emission intensity/efficiency of the degraded sample drops to ∼37% of its initial state before degradation. Nevertheless, the findings demonstrate that the APE sample has a credible tolerance to humidity, oxygen and uninterrupted 100-hour pumping.

### Enhancing the light extraction of APE

**Figure 3.**
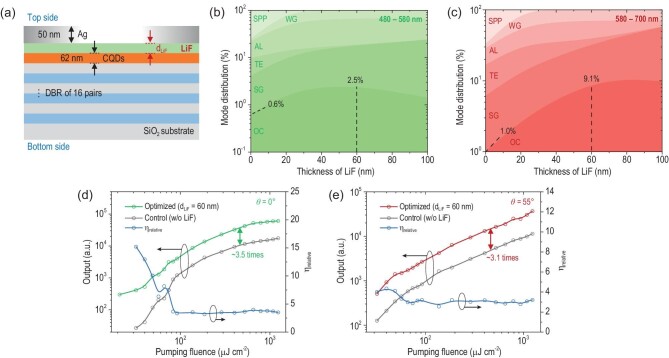
Enhancement of the light extraction of APE. (a) The structure of the cavity consists of an Ag thin film, LiF spacer layer, sandwiched QD layer and the bottom 16-pair Ta_2_O_5_/SiO_2_ DBR. (b, c) Simulated mode distribution of the (b) 480–580 nm and (c) 580–700 nm region. SPP, WG, AL, TE, SG and OC correspond to: surface plasmon polaritons, waveguide in layers except substrate, absorption loss, top emission, waveguide in substrate and out-coupled modes, respectively. (d, e) Emission intensity of the control and optimized samples and the ratio between them at (d) *θ* = 0° and (e) *θ* = 55°.

Although the APE from monodisperse QDs has been well demonstrated, its light extraction efficiency (LEE) should be limited due to the strong quenching effect caused by the loss of metal-induced surface plasmon polaritons (SPPs) when the dipoles in QDs are adjacent to the Ag thin film. To improve the LEE, we introduced a thermal-evaporated LiF spacer layer with thickness of *d_LiF_* between Ag and QDs (see Fig. 3a) to reduce the SPP loss by separating the dipoles from the lossy region. The PLQY of the QDs remained at 94% of its initial value after LiF spacer layer deposition (see Fig. S10), indicating a negligible impact on its emission properties. Considering the APE characteristic, we divided the emission into green (480–580 nm) and red (580–700 nm) parts to evaluate their LEE respectively. The simulated mode distribution, including SPP, waveguide (WG) in layers except substrate, absorption loss (AL), top emission (TE), substrate-guided (SG) and outcoupled (OC, corresponding to the LEE in the bottom side) modes for green and red emission from cavities with various *d_LiF_* ranging from 0 to 100 nm, are illustrated in Fig. 3b and c, respectively. When *d_LiF_* is increased from 0 to 60 nm, the LEE for green (LEE_G_) and red (LEE_R_) emissions increases, from 0.6% and 1.0% to 2.5% and 9.1%, respectively. Specifically, the enhancement of LEE could be primarily attributed to the suppressed SPP modes from 74.9% and 66.9% to 0.2% and 0.2% for red and green emissions, respectively. The simulation of field distribution within the cavity given in Fig. S11 also demonstrates that the ratio of field energy confined in the Ag region (Γ_Ag_) decreases significantly with introduced LiF, which is contributing to the reduction of SPP loss. It is also worth mentioning that the *d_LiF_* of 60 nm is not the optimum for both red and green emissions but a compromise to achieve a relatively higher overall LEE. It is possible to achieve an even higher LEE_R_ of 10.8% if the *d_LiF_* is further increased to 80 nm, but the LEE_G_ should be decreased to 1.9% at the same time. Apart from the LEE enhancement, the introduction of an LiF spacer layer also improves the positive Purcell effect. A detailed discussion can be found in Fig. S12 and the corresponding context.

The emission intensity over *P* of the control and optimized samples, as well as the ratio between them (*η_relative_* = *I_optimized_*/*I_control_*) at *θ* = 0° (green emission) and 55° (red emission), are given in Fig. 3d and e, respectively. The corresponding *P*-dependent emission spectra of the optimized sample can be found in Fig. S13. As shown in Fig. 3e, the optimized sample exhibits a lower *P_th_,_APE_* of ∼21 μJ cm^−2^ compared to that of the control sample (32 μJ cm^−2^) as well as the 1P transition of the QD film (55 μJ cm^−2^). Eighteen APE samples consisting of nine control and nine optimized ones have been fabricated and characterized, revealing great repeatability and uniformity across the samples in terms of their PL wavelength, intensity and *P_th_,_APE_* (see Fig. S14). The decreased *P_th_,_APE_* could be explained by the inhibition of SPP loss (see Fig. 3b and c) and the reduction of Γ_Ag_ from 1.7% to 0.4% with the introduced 60 nm LiF layer. Consequently, the *η_relative_* could be as large as 15.2 at *P* = 32 μJ cm^−2^ due to the different turn-on characteristics of the two samples. When the *P* is above 100 μJ cm^−2^, the *η_relative_* drops to ∼3.5 and remains stable with increased *P*. In contrast, the *η_relative_* curve at *θ* = 55° keeps relatively flat with various *P* as shown in Fig. 3e, which could be attributed to the threshold-less 1S transition of QDs.

### A demo: pixelated cavity for full-color emission

For full-color micro-display, the patterning and pixelation of red, green and blue subpixels are inevitable. For example, a feasible and widely used scheme is to separately couple color-converting green and red QDs with a blue GaN-based micro-LED by either non-radiative or radiative energy transfer, to achieve full-color emission [29]. However, the two-step direct patterning of red and green QDs not only requires high-precision alignment, but also, in some cases, introduces irreversible deterioration of the optical properties of QDs [30,31]. Although there have been many studies reporting polychromatic emission from monodisperse QDs [9–22], none of them have simultaneously achieved separation of different colors and full-color pixelation using monodisperse QDs (see Table S1).

For the APE sample, the green emission at 0° is attributed to the coupling of 1P-band emission into the sideband mode of the cavity. Without a complete cavity, the QDs would emit red light in all directions. Namely, it is possible to define the red and green subpixels by patterning the top Ag thin film, which is a critical component of the entire cavity, through a simple one-step evaporation process using FMM.

**Figure 4.**
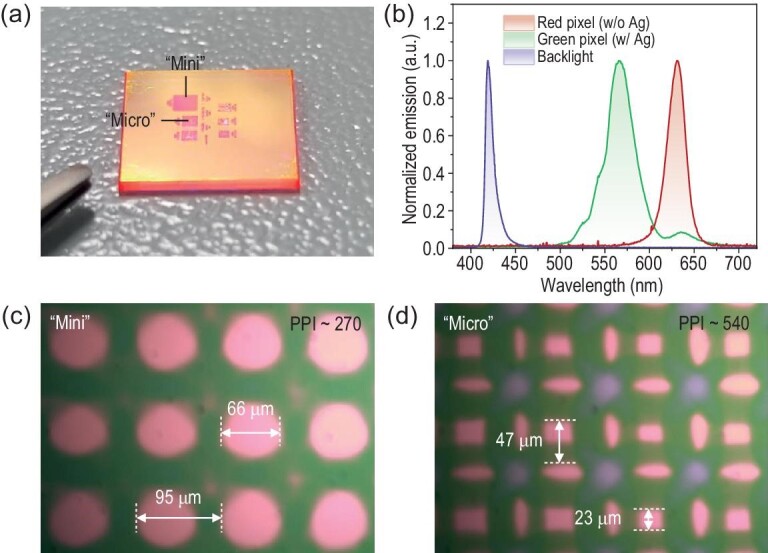
A demo: pixelated APE for full-color emission. (a) The APE sample with patterned Ag thin film in different regions. Two regions are marked as ‘Mini’ and ‘Micro’. The photograph was taken from the front side of the sample. (b) Emission spectra (*θ* = 0°) of the green (with Ag thin film) and red (without Ag thin film) subpixels in the patterned region of the sample under *P* of 30 μJ cm^−2^. (c, d) Microscopic images of the ‘Mini’ and ‘Micro’ regions under excitation of 355 nm pulse laser and 420 nm LED backlight. The spectra and images shown in (b–d) were taken from the back (substrate) side of the sample.

Here, we have fabricated a sample with patterned Ag thin film as shown in Fig. 4a, in which two regions marked ‘Mini’ and ‘Micro’, corresponding to a pixel size above and below 50 μm, are pointed out, respectively. As expected, green or red emission can be detected at *θ* = 0° from the regions with or without Ag thin film, in the same sample, under pulse excitation of 30 μJ cm^−2^. The corresponding spectra are illustrated in Fig. 4b. Notably, although the emission spectrum from red subpixels generally aligns with the spontaneous emission as shown in Fig. 1a, the green ones exhibit a larger FWHM than the un-patterned sample, as shown in Fig. 2e. Additionally, the green subpixels show another emission peak at ∼630 nm. The difference could be attributed to our characterization set-up (see Fig. S2). The light collection spot size is ∼200 μm while the maximum collection angle of the 50 × objective (with NA = 0.45) is 26.7°. Consequently, it is inevitable to collect the signal from multiple subpixels emitting light in different directions simultaneously, including some red ones that are responsible for the peak at 630 nm. Besides, the crosstalk issue could hopefully be addressed by inserting light-absorbing materials among them [32].

The microscopic images of the ‘Mini’ and ‘Micro’ regions under excitation of 355 nm pulse laser and 420 nm LED backlight are given in Fig. 4c and d, respectively. The image of the ‘Mini’ region (Fig. 4c) shows that a pixel array consisting of red and green subpixels has been successfully constructed by patterning the Ag thin film. The size of the red subpixels is ∼66 μm, and the pixel pitch between them is ∼95 μm, resulting in a pixel density of 270 pixels per inch (PPI). Furthermore, by minimizing the pattern in the FMM, a pixel size as small as 23 μm and pixel pitch of ∼47 μm could been achieved (see Fig. 4d), corresponding to a pixel density of 540 PPI. The presence of the blue color in Fig. 4d can be attributed to limitations encountered during the laser patterning process for the smaller features. The patterns within the ‘Micro’ region proved challenging to define precisely using our 1064 nm fiber laser marking machine. Consequently, non-uniformities arose in the Ag film thickness, particularly in the blue pixel regions where the film became ultrathin (<5 nm). As illustrated in Fig. S4e and f, the emission intensity of the APE sample with a 5 nm Ag film shows a significant reduction (54% and 69% at 0° and 55°, respectively) compared to that of standard 50 nm Ag film. This decrease in APE intensity, coupled with the suppression of intrinsic red emission from QDs due to metal-induced SPP loss, allows the reflected 420 nm blue backlight to become the dominant light source. This ultimately leads to the blue-purple appearance observed in Fig. 4d. Besides, the colors shown in Fig. 4c and d may be distorted due to the light filter in our microscope system. Nevertheless, it is evident that a full-color micro-pixel array can be achieved using monodisperse QDs as color-converting materials.

### Quasi-continuous-wave pumped APE

Although we have demonstrated ps-pulse pumped APE as discussed earlier, its practicality is still questionable due to the high cost and bulkiness of the pumping laser source. Hence, it is necessary to investigate whether it is possible to stimulate the APE using more accessible and compact LDs or LEDs whose power density and pulse duration is relatively limited. Here, we use another 393 nm laser with 29 ns pulse duration and 50 kHz repetition rate as the new pumping source. As the biexciton lifetime of CdSe-based QDs is typically ∼1 ns or smaller [8], the 29-ns pump can actually be considered quasi-cw. As previously reported, the lasing threshold (*E_th, las_* in W cm^−2^, the average power density within a laser pulse) of quasi-cw pumped colloidal QD lasers should be approximately consistent with the real cw-pumped ones [33]. Besides, the quasi-cw pumping mode could avoid sample breakdown by reducing heat accumulation with smaller integrated power compared to the cw mode [9,34].

**Figure 5.**
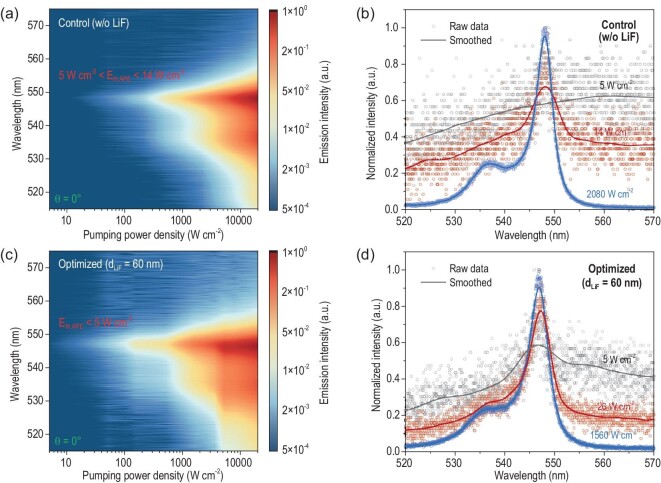
Characterization of the quasi-cw laser pumped APE. (a) Pumping power density dependent emission spectra of the control sample (w/o LiF) at *θ* = 0°. (b) Raw data and smoothed curves of the emission spectra of the control sample at *θ* = 0° under a pumping power density of 5, 14, 2080 W cm^−2^. (c) Pumping power density-dependent emission spectra of the optimized sample (with 60 nm LiF) at *θ* = 0°. (d) Raw data and smoothed curves of the emission spectra of the optimized sample at *θ* = 0° under a pumping power density of 5, 26, 1560 W cm^−2^.

The emission spectra of the control sample in the vertical direction (*θ* = 0°), under pumping intensity *E* ranging from 5 to 20 000 W cm^−2^, are given in Fig. 5a. The first peak at ∼547 nm emerges when the *E* is relatively small. With increased *E*, the emission at *θ* = 0° tends to be stronger and broader, along with another peak appearing at ∼535 nm. The raw data obtained from the spectrometer and the corresponding smoothed curves when *E* = 5, 14 and 2080 W cm^−2^ are presented in Fig. 5b. When *E* is 5 W cm^−2^, the raw data (black points) appear chaotic, with no discernible peak in the smoothed curve in black. When *E* reaches 14 W cm^−2^, a distinct peak at 547 nm can be observed in the smoothed curve in red. Therefore, the APE threshold (*E_th_,_APE_*) for the control sample should be between 5 and 14 W cm^−2^ (or, *P_th_,_APE_* between 100 and 280 μJ cm^−2^ @ 29-ns pulse). Further increasing the *E* to 2080 W cm^−2^, the signal-to-noise ratio (SNR) of the acquired spectrum (in blue) can be significantly improved so that it is higher than 100:1, revealing a clear FWHM of 4.1 nm.

The *E*-dependent emission spectra of the optimized sample, with a 60 nm LiF spacer layer at *θ* = 0°, are also characterized, as shown in Fig. 5c. Similar to the control sample, the optimized one exhibits a single peak at 547 nm with a small *E* and experiences broadening in the spectrum with increased *E*. As mentioned before, the optimized sample showed a decreased *P_th_,_APE_* from 32 to 21 μJ cm^−2^ with reduced SPP loss. Here, as shown in Fig. 5d, the smoothed curve (in black) of the emission spectrum from the optimized sample under *E* = 5 W cm^−2^ exhibits an evident peak at 547 nm, indicating an *E_th_,_APE_* below 5 W cm^−2^. It should be noted that the upper limit of the *E_th_,_APE_* could be even lower, but it is hard to determine an accurate *E_th_,_APE_* with our laser power-meter, which has limited sensitivity. Nevertheless, the acquired experiment results have already demonstrated the lower *E_th_,_APE_* of the optimized sample compared to the control one. It is worth mentioning that the 29 ns pulse width is the longest among all reported pump sources that have successfully induced multi-exciton polychromatic emission in colloidal QDs. Additionally, the threshold of 5 W cm^−2^ ranks among the lowest reported for similar phenomena (refer to Table S1 for a detailed comparison) [9–22].

We should note that such a low *E_th_,_APE_* below 5 W cm^−2^ indicates that the APE could be stimulated even by accessible compact LD and LED, in principle. For example, 455 nm LD (no. NDB7Y75) packaged with 1.6 mm aperture from Nichia could support an output power over 5 W at cw mode, corresponding to an *E* higher than 249 W cm^−2^ at its output point, which is adequate to excite APE from our sample. Also, it was reported that the GaN mini-LED could provide an *E* over 400 W cm^−2^ with intense current injection [35]. These findings reveal the potential for the application of LED- and/or LD-pumped APE for practical full-color micro-displays. As shown in Fig. S15, when combined with a 455 nm LD, the APE enabled a monodisperse QD-based micro-display system that could support an National Television Standards Committee (NTSC) and BT.2020 coverage of 112% and 84%, respectively.

## DISCUSSION

This study has demonstrated a unique APE phenomenon of monodisperse CdSe-based QDs, namely, that the QDs can emit red and green light in different directions when they are stimulated by a pump laser into multi-excitonic states and then integrated into an angle-dependent wavelength-selective cavity. The cavity structure was then optimized to improve the intensity of red and green emissions by 2.1 and 2.5 times, respectively, and lower the APE threshold by 34%. This was achieved by introducing a LiF spacer layer to reduce the surface plasmon loss and enhance the Purcell effect. Next, using FMM in the thermal evaporation process, we have fabricated a patterned red and green micro-pixel array with a pixel size as small as 23 μm by simply patterning the Ag thin film, which is a key component of the entire cavity for supporting APE. Surprisingly, we found that the APE could be stimulated above a threshold as low as ∼5 W cm^−2^ using a quasi-cw laser, indicating its compatibility with commercially available LEDs and LDs, and therefore, its potential practical application.

Notably, the APE reported here still requires an external optical pumping source. It is worth considering the possibility of integrating a current injection structure within the cavity to achieve electrically driven APE from QDs. As the electrically driven multi-excitonic amplified spontaneous emission (ASE) from QDs has been demonstrated recently [10], it is also theoretically feasible to achieve APE in a similar structure since the APE threshold under quasi-cw pumping is much lower than the reported colloidal QD ASE/lasers.

Moreover, the innovative approach of harnessing monodisperse QDs with multi-excitonic behavior under low pumping intensity has exciting prospects for further investigation of polychromatic diode-pumped colloidal QD lasers. For this purpose, the Purcell effect and surface plasmon loss caused by the metal adjacent to QDs should be well managed to facilitate efficient light amplification. Also, improving the cavity structure is necessary to enhance the quality factor of resonant modes to support low-threshold lasing.

## MATERIALS AND METHODS

### Fabrication of the APE sample

First, the 16-pair bottom Ta_2_O_5_/SiO_2_ DBRs with reflectance peak at 635 nm are deposited on quartz substrate by ion-assisted e-beam evaporation. The thickness of each Ta_2_O_5_ and SiO_2_ layer is 73 nm and 109 nm, respectively. After 30-min ultrasonic cleaning and 5-min plasma cleaning of the DBRs-on-substrate, the CdZnSe/ZnSe/ZnCdS QDs dissolved in octane with a concentration of 50 mg/mL are spin-coated onto the bottom DBRs at 3000 r/min for 40 s and baked at 90°C for 5 min in a glove box to form a close-packed QD film with a thickness of 62 nm. After that, the Ag thin film is thermally evaporated onto the QD film with a deposition rate of ∼0.1 Å/s. The pressure in the chamber is carefully controlled to below 3 × 10^−4^ Pa. During characterization, the sample is flipped to emit light from its substrate side. The emission from the Ag mirror side is weak due to the low transmittance (<3%) of the Ag mirror. The fabrication flow of the APE sample is illustrated in Fig. S6.

### PL characterization

For ps-pulse excitation, a diode-pumped solid-state pulsed laser, MPL-FN-355 from CNI, emitting at 355 nm with a pulse width of 300 ps and a repetition rate of 100 Hz, is used as the pump source. The pumping fluence is adjusted by two laser power attenuators (Optogama LPA-M 355 nm) in series. The spectrometers coupled to the system are Ocean Optics USB2000+ and AvaSpec ULS4096CL EVO. For quasi-cw pulse excitation, the pump laser is CNI TUN-TiA-393-408 at 393 nm with a pulse width of 29 ns and repetition rate of 50 kHz. The spectrometer is Horiba iHR 550 with Syncerity CCD. Detailed information on the PL characterization set-up can be found in Fig. S2.

## Supplementary Material

nwae311_Supplemental_Files
