# Supplementary material for: Low-threshold anisotropic polychromatic emission from monodisperse quantum dots
Source: Natl Sci Rev. 2024 Sep 5;12(2):nwae311. doi: 10.1093/nsr/nwae311 (PMC11745159; doi:10.1093/nsr/nwae311)
Supplement: nwae311_Supplemental_Files [file nwae311_supplemental_files.zip › Revised Supplementary Information.docx]

Supplementary Information for

**Low-threshold Anisotropic Polychromatic Emission from Monodisperse Quantum-dots**

Yangzhi Tan *et al.*

*Corresponding author. Email: wangk@sustech.edu.cn, hwchoi@hku.hk

**This file includes:**

Note S1

Figs. S1 to S15

Table S1

References (1 to 5)

**Other Supplementary Materials for this manuscript include the following:**

Movie S1

Note S1. Derivation of ideal threshold for high-energy 1S_e_-1S_lh_ and 1P_e_−1P_hh_ transitions

To quantify the number of excitons per QD (<*N*>) required to achieve population inversion for higher-energy transitions, we employ an electronic-state model of spherical QD, as illustrated in Figure 1B. The model accounts for the selection rule that optical transitions are only permitted between electron and hole states with identical quantum numbers (e.g., 1S_e_-1S_hh_, 1S_e_-1S_lh_ and 1P_e_−1P_hh_). To satisfy the threshold for a specific transition coupling the *i_e_* electron level and the *j_h_* hole level, the following condition must be met:

*f_e,i_* + *f_h,j_* = 1 (1)

where *f*_e,_*_i_* and *f*_h,_*_j_* are single-state occupation factors, which can be determined by *f*_e,_*_i_ = <N_e,i_*>/*g_e,i_* and *f_h,j_* = *<N_h,j_*>/*g_h,j_*. The *<N_e,i_*> and *<N_h,j_*> represent the total number of electrons (holes) occupying a specific level, and *g_e,i_* and *g_h,j_* denote the corresponding level degeneracy (the number of possible spin states for a given energy level). The *g* of the S and P levels are 2 and 6 for CdSe-based QDs, respectively. Assume that the QDs are neutral without charging, the QD electron occupancy is equal to that of hole: *<N_e_*> = *<N_h_*> = *<N*>. For QDs with *<N*> ≤ 2, all carriers reside in the 1S states, and the 1S_e_ and 1S_hh_ occupation factors are *f*_e,_*_1Shh_* = *f*_e,_*_1Se_* = *<N*>/*g_S_* = *<N*>/2. Therefore, the threshold of population inversion in band-edge 1S_e_-1S_hh_ transition can be derived from:

*f_e,1Se_ + f_h,1Shh_ = <N_e_*>/*2 + <N_h_*>/*2 = <N*> = 1 (2)

Similarly, one can derive the threshold for 1S_e_-1S_lh_ (marked as 1S’) and 1P_e_-1P_hh_ (marked as 1P) transitions from

*f_e,1Se_ + f_h,1Slh_ = <N_e_>*/*2 +* (*<N_h_>* - 2)/2 = *<N>* - 1 = 1 (3)

*f_e,1Pe_ + f_h,1Phh_* = (*<N_e_>* - 2)/6 + (*<N_h_>* - 4)/6 = *<N>*/3 - 1 = 1 (4)

The resulting threshold for 1S’ and 1P transitions are <*N*> = 2 and 6, respectively.


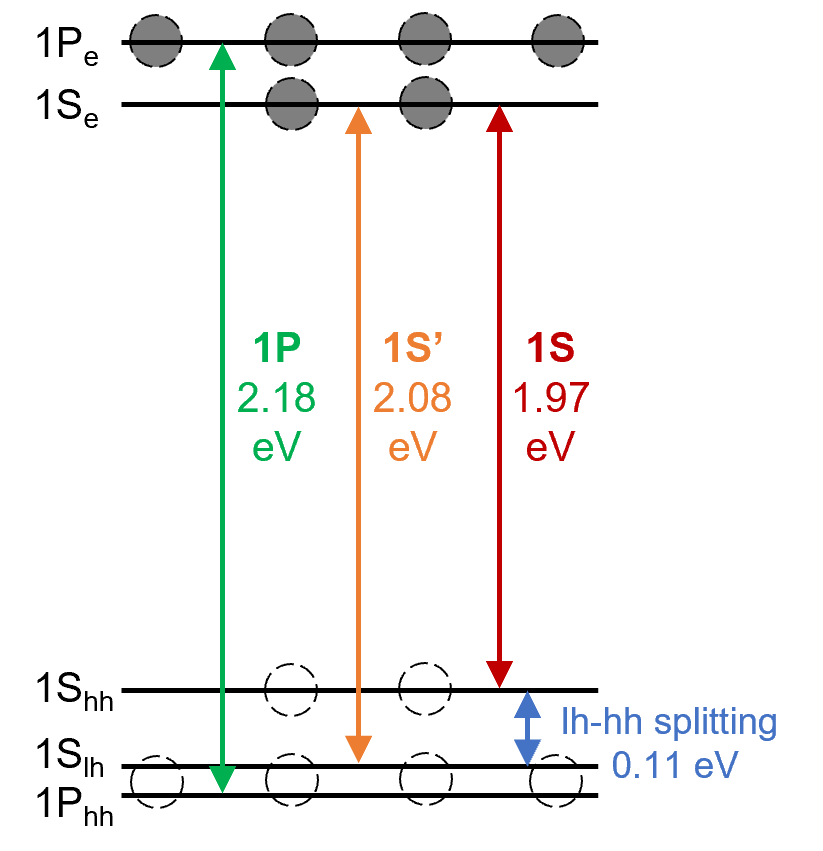


Figure S1. Size distribution of the monodisperse QDs.

**(A) - (C)** The transmission electron microscopy (TEM) images of the QDs used in this work, the scale bar is 50 nm.

**(D)** The QDs size distribution shows a mean value (*μ*) of 12.6 nm and a standard deviation (*σ*) of 1.5 nm by Gaussian fit.


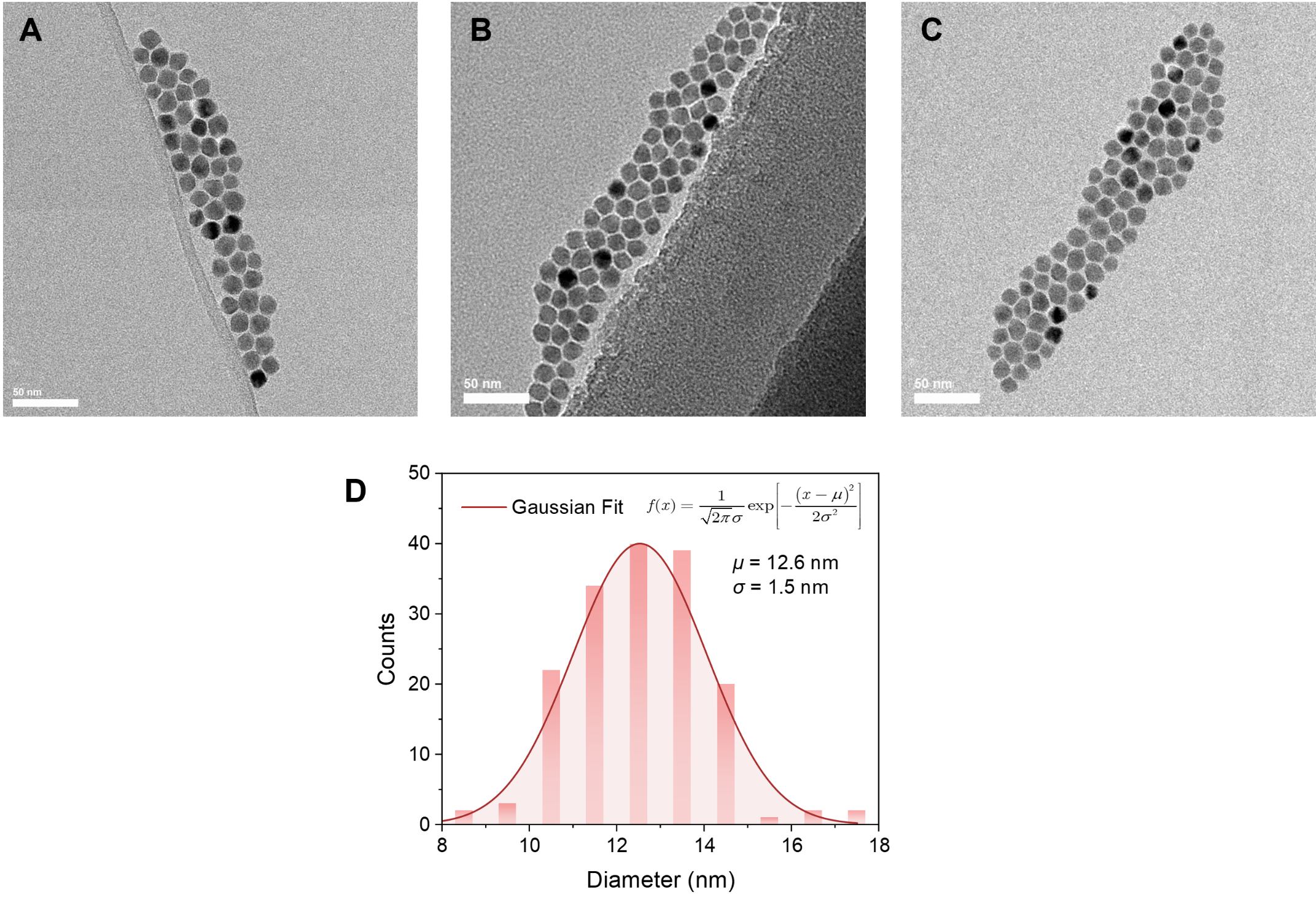


Figure S2. Photoluminescence (PL) characterization setup

**(A)** The structure of PL characterization setup. The objective in the Z-axis is the primary component for signal collection due to the relatively weak edge emission from the APE sample. The light collection spot size for this setup using 50× objective is approximately 200 μm. The numerical aperture (NA) of the 50× objective in the setup is 0.45, corresponding to a maximum collection angle of 26.7°.

**(B)** Schematic of the PL characterization of the APE sample. During characterization, the APE sample is flipped to emit light from its substrate side. The emission from the Ag top mirror side is weak due to the low transmittance (< 3%) of Ag mirror. By adjusting the stage rotation angle, we can collect the angle-dependent PL signal from the APE sample.


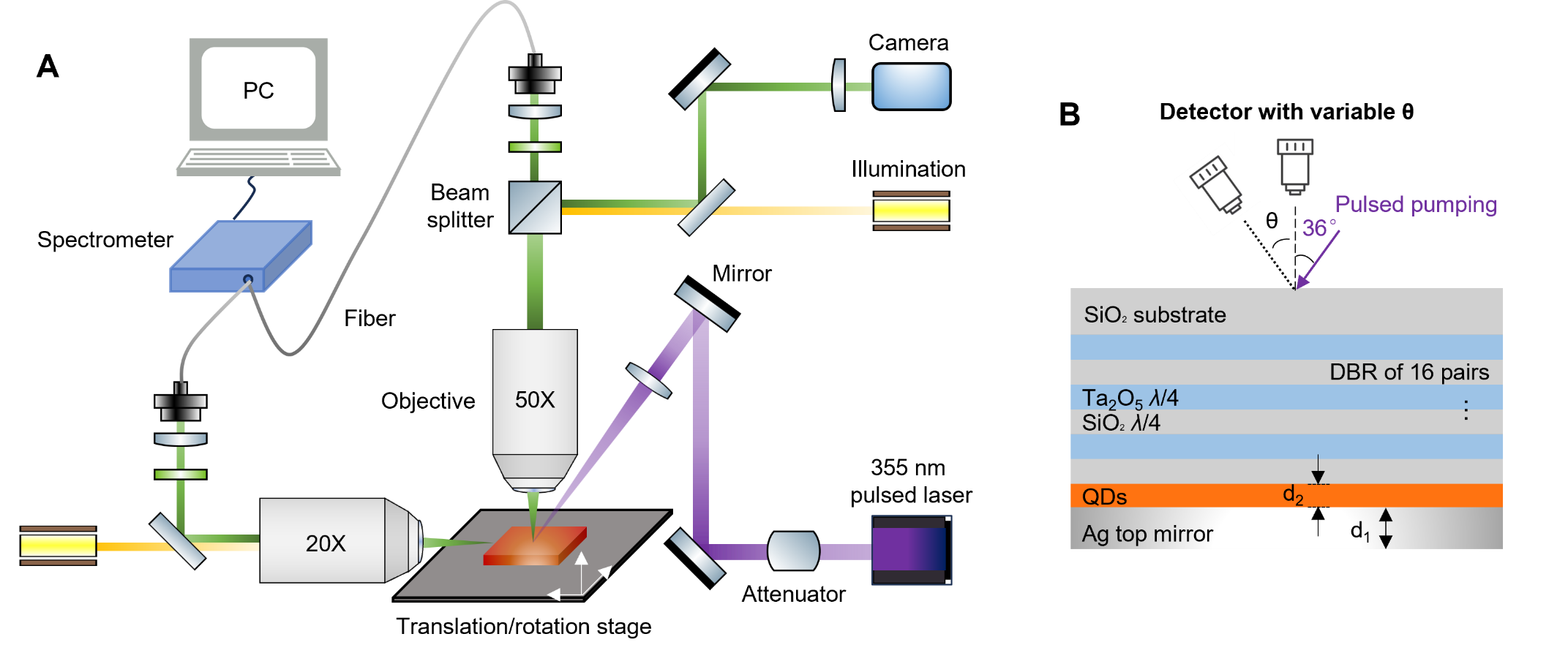


Figure S3. The random lasing characteristics of QDs

**(A)** The emission spectra of the QDs film on quartz substrate under pulse pumping fluence (*P*) from 17 μJ cm^-2^ to 315 μJ cm^-2^. The pumping light is focused on the edge of the sample. Below the lasing threshold, the QDs exhibit spontaneous emission at 630 nm (1.97 eV) with FWHM of about 25 nm, corresponding to the 1S transition. When the *P* comes to 26 μJ cm^-2^, some lasing modes at around 640 nm (1.94 eV) are observed. The red-shift from the spontaneous emission to ASE/lasing is attributed to the carrier-induced Stark effect that describes the Coulomb interaction between the initially generated exciton and the other electron to be excited [1]. In type I CdSe/ZnS QDs used in our work, the Coulomb attractive force between excitons lowers the bandgap when the per-dot exciton occupancy <*N*> is larger than 1, leading to the red-shift phenomenon as shown here. Further enhance the *P* to 166 μJ cm^-2^, other lasing modes at around 603 nm (2.06 eV) emerge, corresponding to the 1S’ transition as illustrated in Figure 1b. It should be noted that the test range of the high-precision spectrometer used here is about 597-700 nm, some possible lasing modes beyond that range are undetectable.

**(B)** The partial emission spectrum of 635-650 nm of the sample under *P* of 133 μJ cm^-2^. Some random lasing modes can be extracted from the spectrum by peak fitting. It is obvious that the free space range (FSR) between different modes is not uniform, indicating the lasing is from the random scattering instead of the engineered cavity. We marked one of the modes at 644.60 nm with FWHM of about 0.30 nm, corresponding to a Q-factor of 2140.

**(C)** The emission intensity and FWHM versus pumping fluence. The lasing action is evidenced by the non-linear growth of the emission intensity over *P* as well as the sudden decrease of the FWHM near the lasing threshold of around 26 μJ cm^-2^. The coupling efficiency of the spontaneous emission into cavity mode (*β*) is estimated to be about 0.30 [2].


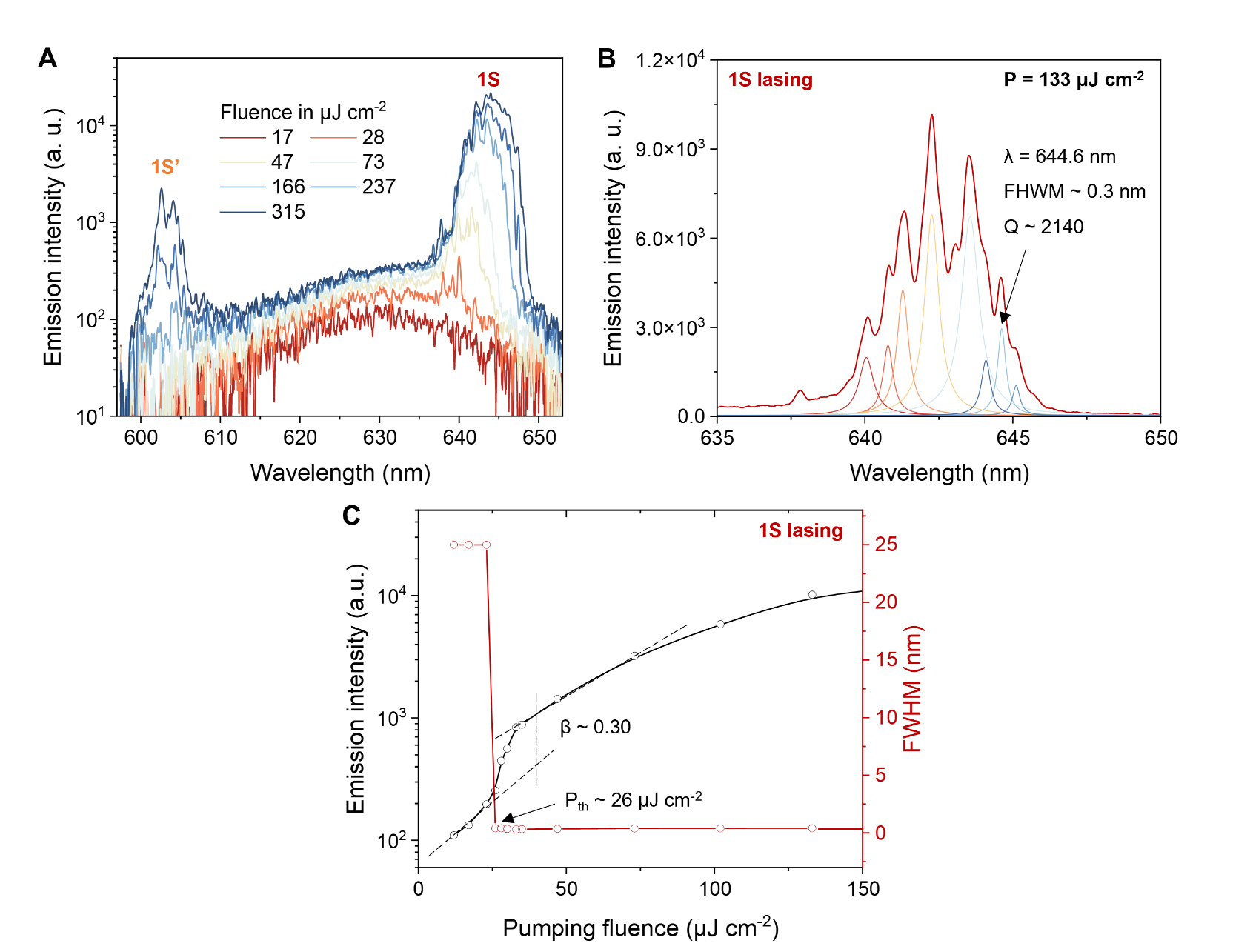


Figure S4. Structure optimization of the control APE sample

The simulated reflectance spectra of Ag thin film with **(A)** various thickness and **(B)** thickness of 50 nm on quartz substrate. A 50 nm Ag film thickness yields reflection beyond 94% for both red and green light.

The simulated emission spectra **(C)** at 0° and **(D)** at 55° of cavity-coupled QDs with fixed Ag thickness of 50 nm and various QD thickness. Generally, increasing the QD thickness leads to a decrease in green emission intensity and an increase in red emission intensity. A QD thickness of 62 nm was determined to be a suitable compromise, balancing the emission intensities of red and green emission while minimizing parasitic peaks.

The simulated emission spectra **(E)** at 0° and **(F)** at 55° of cavity-coupled QDs with fixed QD thickness of 62 nm and various Ag thickness. Overall, both red and green emission intensities increase with increasing Ag thickness. However, the APE intensity approaches saturation when the Ag thickness is larger than 50 nm. Additionally, for Ag thicknesses exceeding 50 nm, a 575 nm parasitic peak appears in the red emission at 55°, compromising the color purity of the red emission.


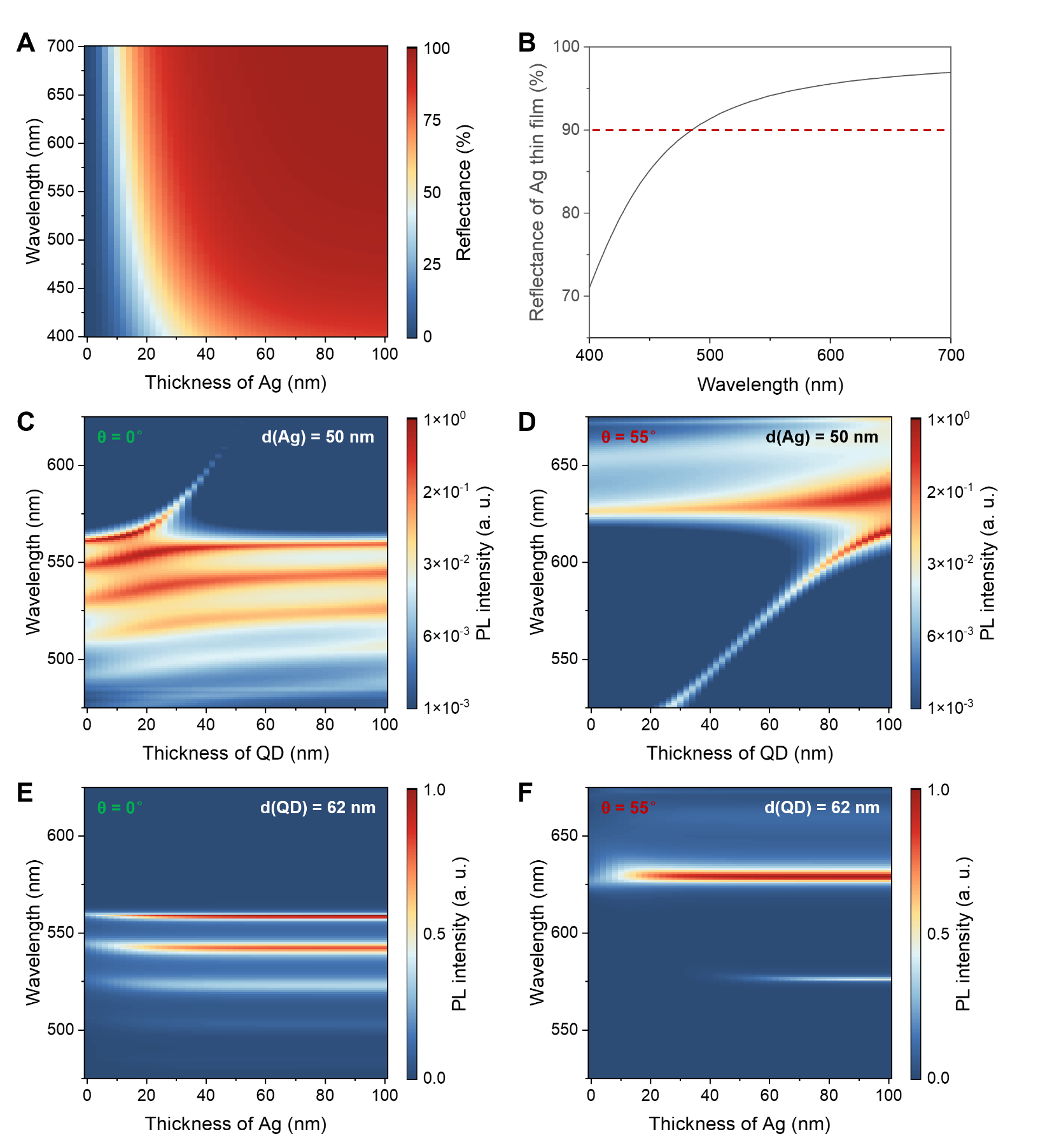


Figure S5. The material refractive indices and characterization of the DBR

**(A)** The real (*n*) and imaginary (*k*) parts of the refractive indices of QDs (upper) and Ag (lower).

**(B)** The upper figure gives the *n* of Ta_2_O_5_ and SiO_2_ (upper). The lower gives the simulated and characterized transmittance of the 16-pair DBR consists of Ta_2_O_5_ and SiO_2_. Each pair contains 73 nm Ta_2_O_5_ and 109 nm SiO_2_. The targeted center wavelength of maximum reflectance of normal incidence is 635 nm, calculated by *λ* = 4*n*_Ta2O5_*d*_Ta2O5_ = 4*n*_SiO2_*d*_SiO2_.


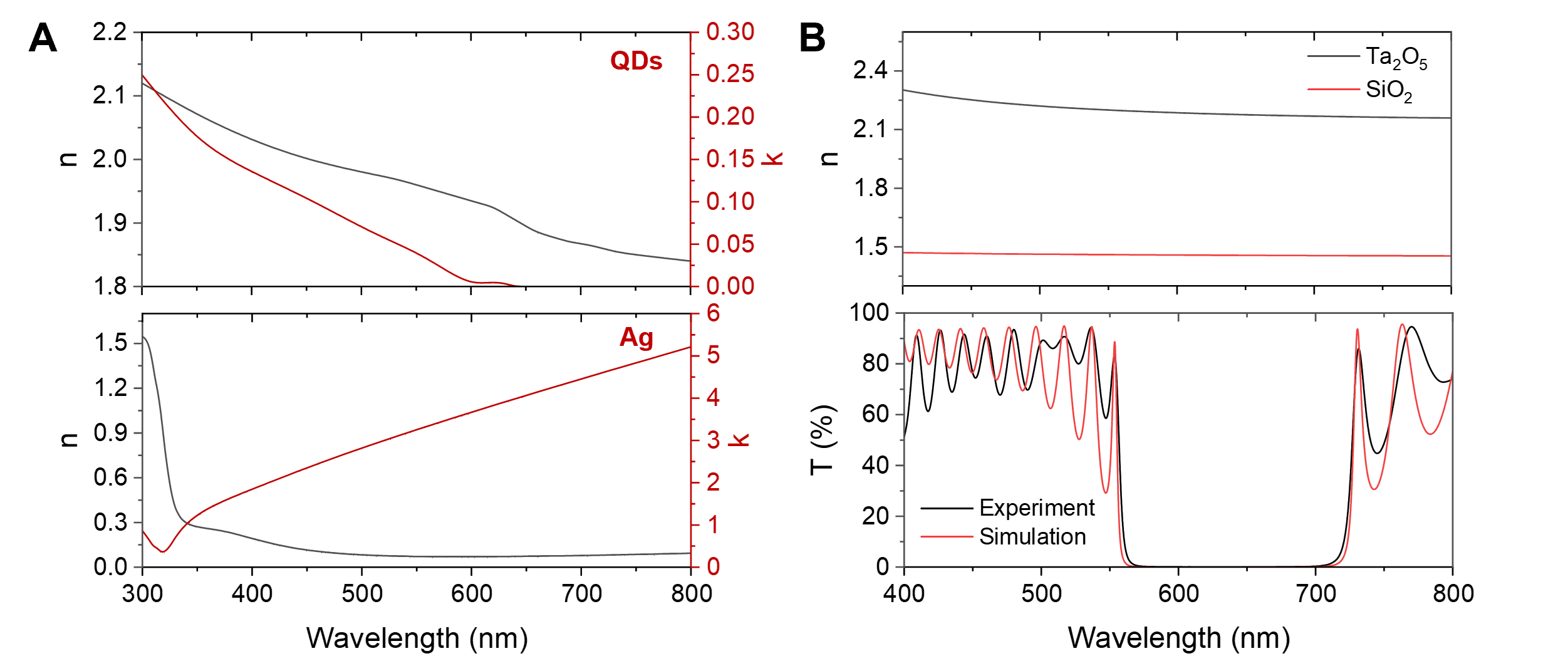


Figure S6. Fabrication process of the APE sample.


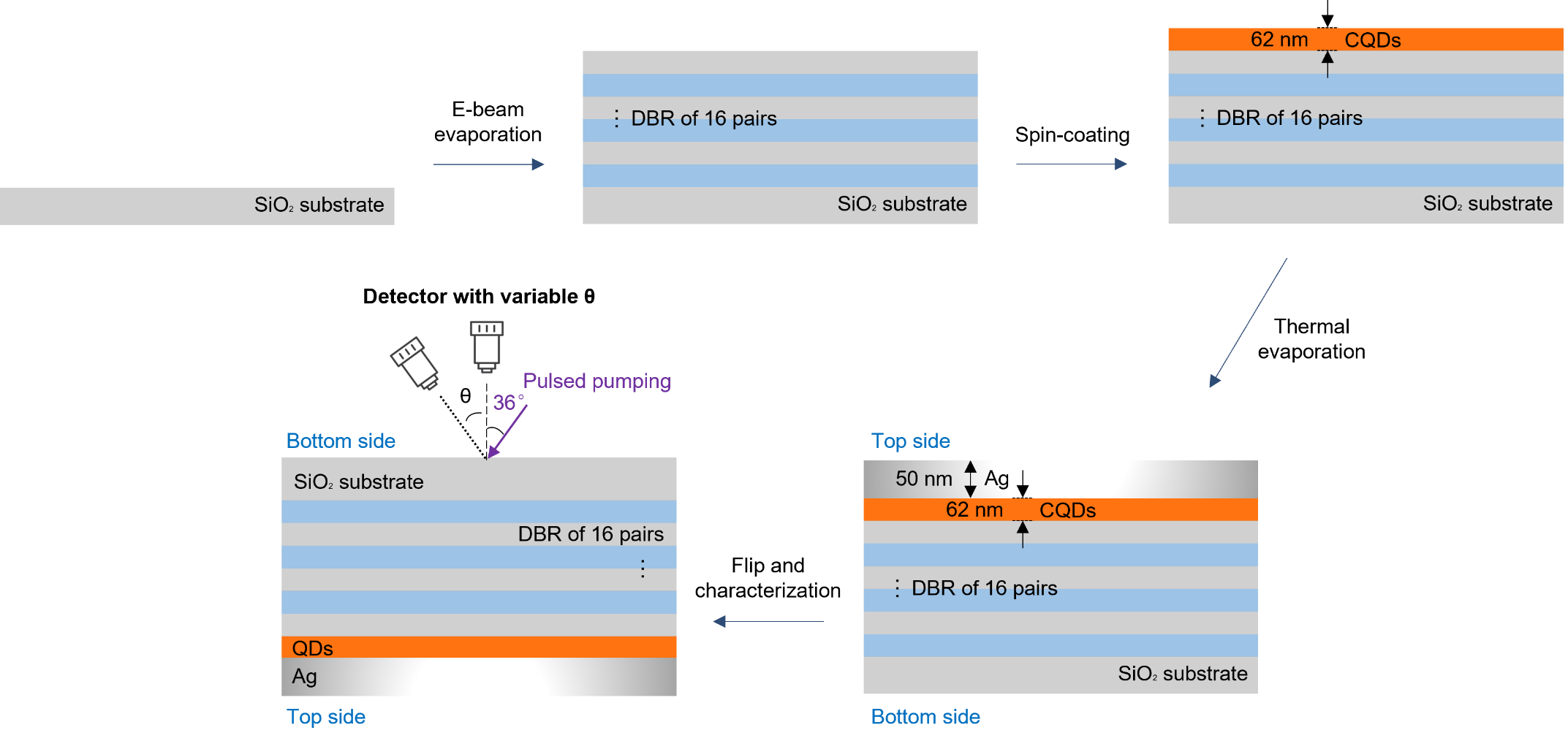


First, the 16-pair bottom Ta_2_O_5_/SiO_2_ DBRs with reflectance peak at 635 nm are deposited on quartz substrate by ion-assisted e-beam evaporation. The thickness of each Ta_2_O_5_ and SiO_2_ layer are 73 nm and 109 nm, respectively. After a 30-minute ultrasonic cleaning and 5-minute plasma cleaning of the DBRs-on-substrate, the CdZnSe/ZnSe/ZnCdS QDs dissolved in octane with concentration of 50 mg/mL were spin-coated onto the bottom DBRs at 3000 rpm for 40 s and baked at 90°C for 5 minutes in glove box to form a close-packed QDs film with thickness of 62 nm. After that, the Ag thin film is thermal evaporated onto the QDs film with deposition rate of about 0.1 Å/s. The pressure in the chamber is carefully controlled to below 3 × 10^-4^ Pa. During characterization, the sample is flipped to emit light from its substrate side. The emission from the Ag mirror side is weak due to the low transmittance (< 3%) of Ag mirror.

Figure S7. The CIE coordinates versus emission angle of the APE from cavity-coupled QDs


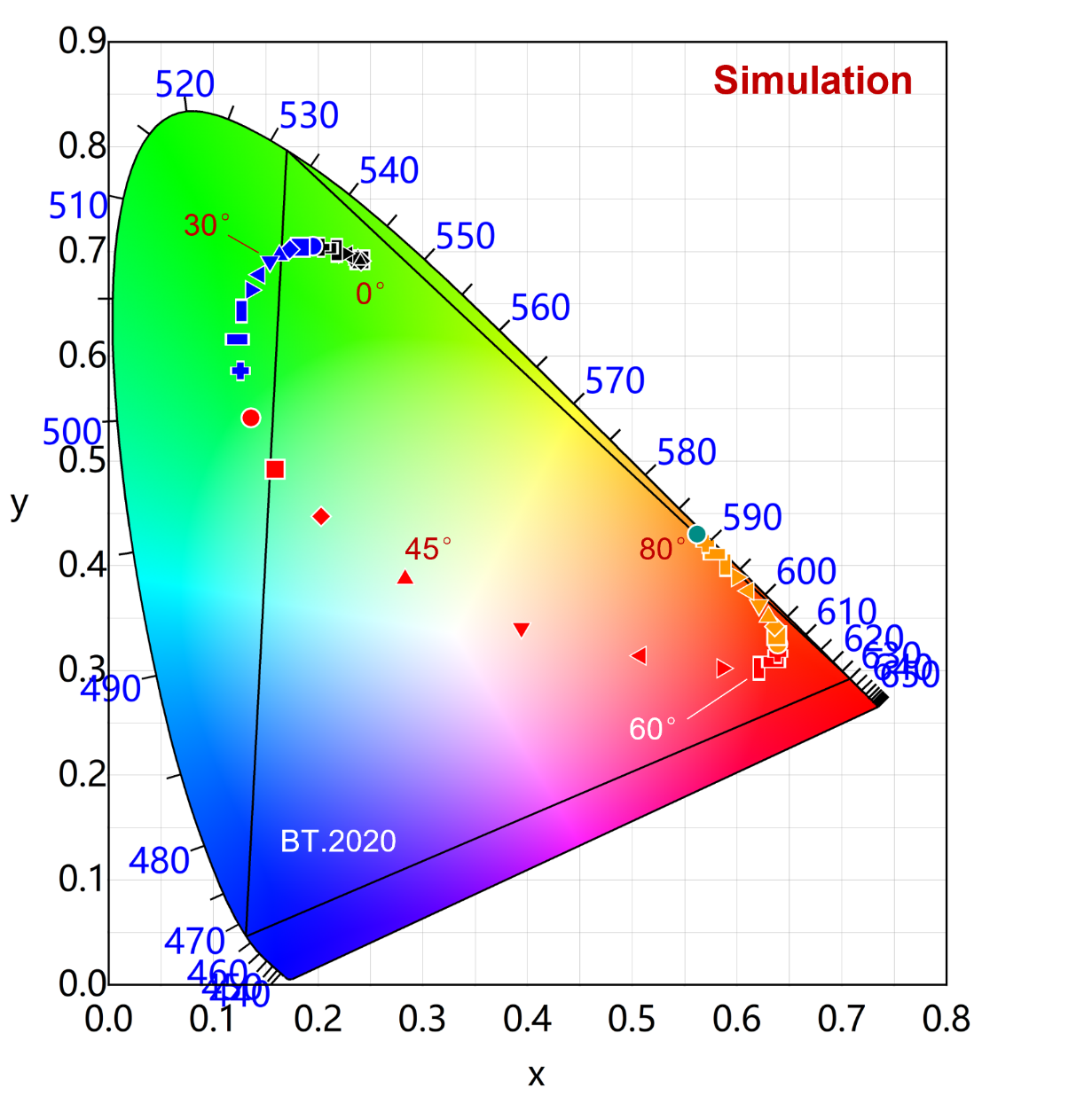


The CIE coordinates corresponding to the simulated emission spectrum of cavity coupled QDs with various emission angles from 0 to 80°. The coordinates of some certain angles are given below.


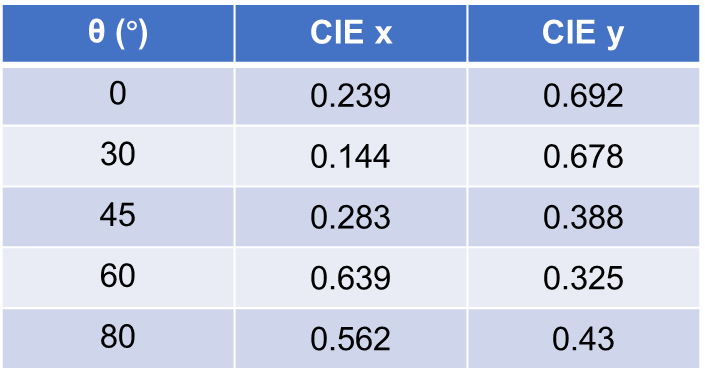


Figure S8. Operational stability of the APE control sample over 100 hours


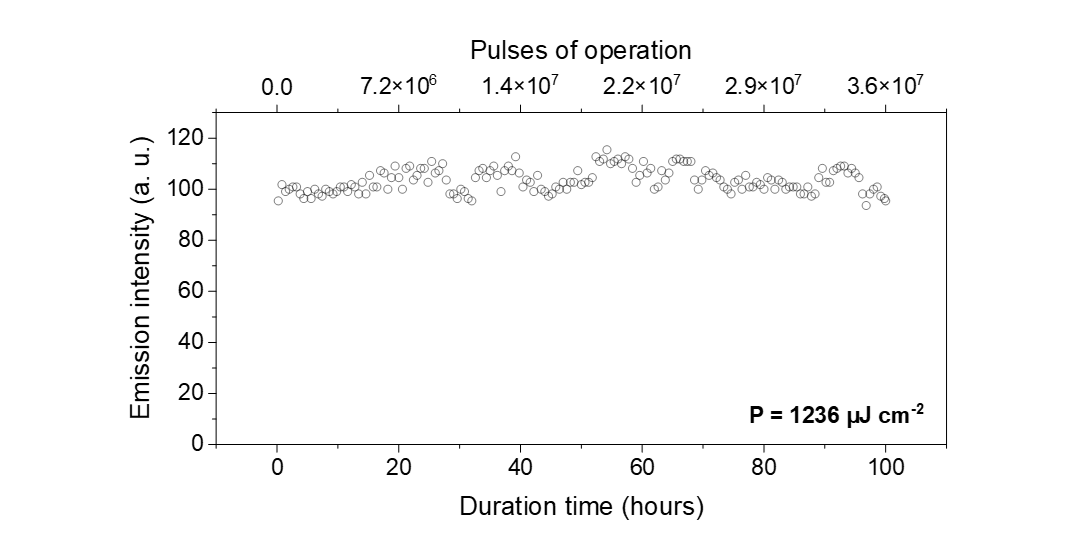


The variation of green emission intensity at 0° for the control sample without LiF spacer layer under a pumping intensity of 1236 μJ cm^-2^ over 10 hours of continuous operation. The repetition rate of the 355 nm pump laser is 100 Hz.

Figure S9. The comparison of the APE characteristic of fresh and degraded samples

**(A)** The emission spectra (*θ* = 0) of fresh cavity coupled QDs without LiF spacer over pumping fluence.

**(B)** The emission spectra (*θ* = 0) of degraded cavity coupled QDs without LiF spacer over pumping fluence. The sample was pumped under a *P* of 1236 μJ cm^-2^ for 24 hours and subsequently placed in atmosphere with a constant temperature of 22 °C and humidity of about 70% for 155 days without any encapsulation.

**(C)** The relative emission intensity (*θ* = 0) of the fresh and degraded samples over pumping fluence.


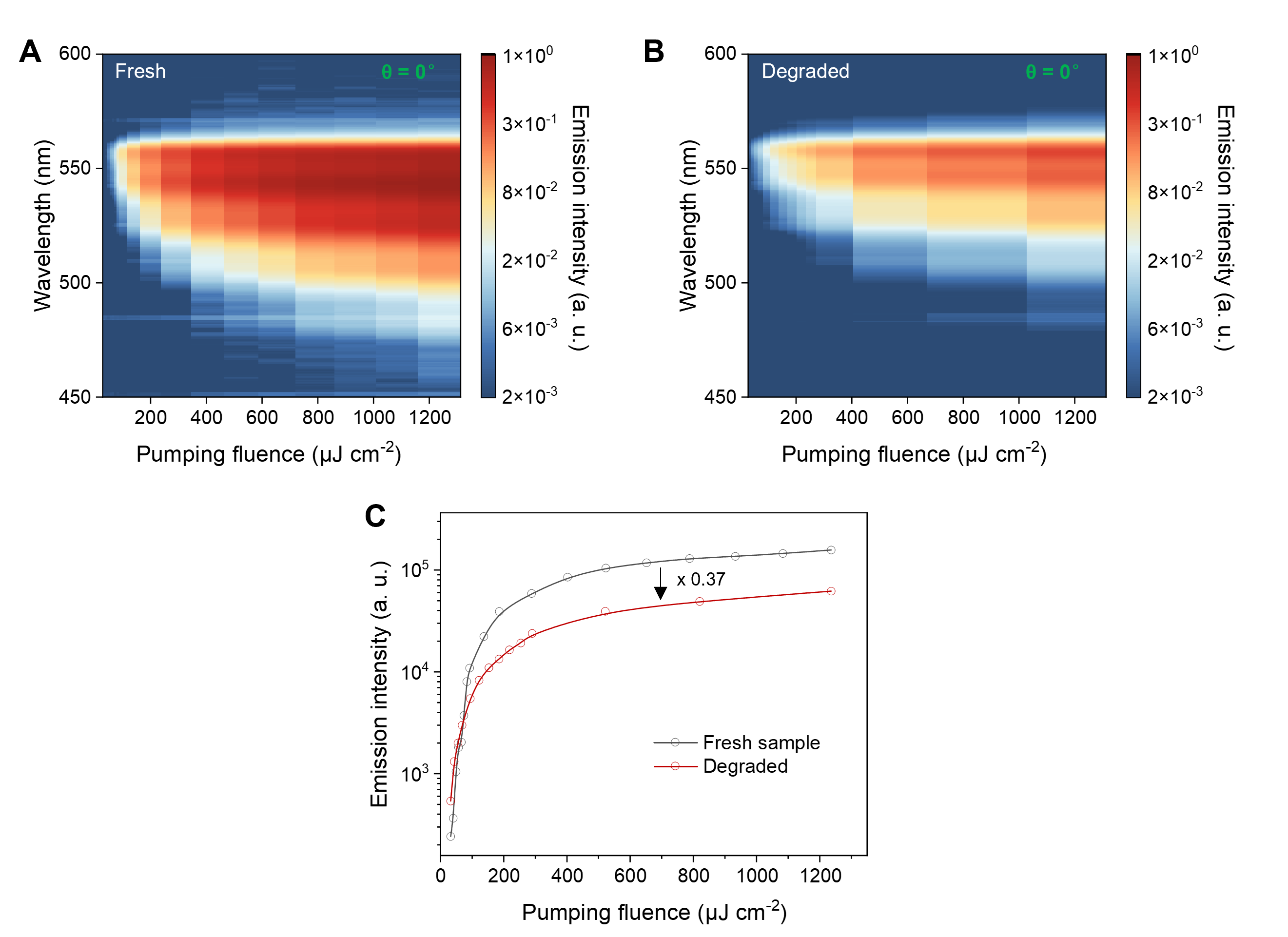


Figure S10. The comparison of photoluminescence quantum yield (PLQY) of QDs post-deposited with different spacer layers


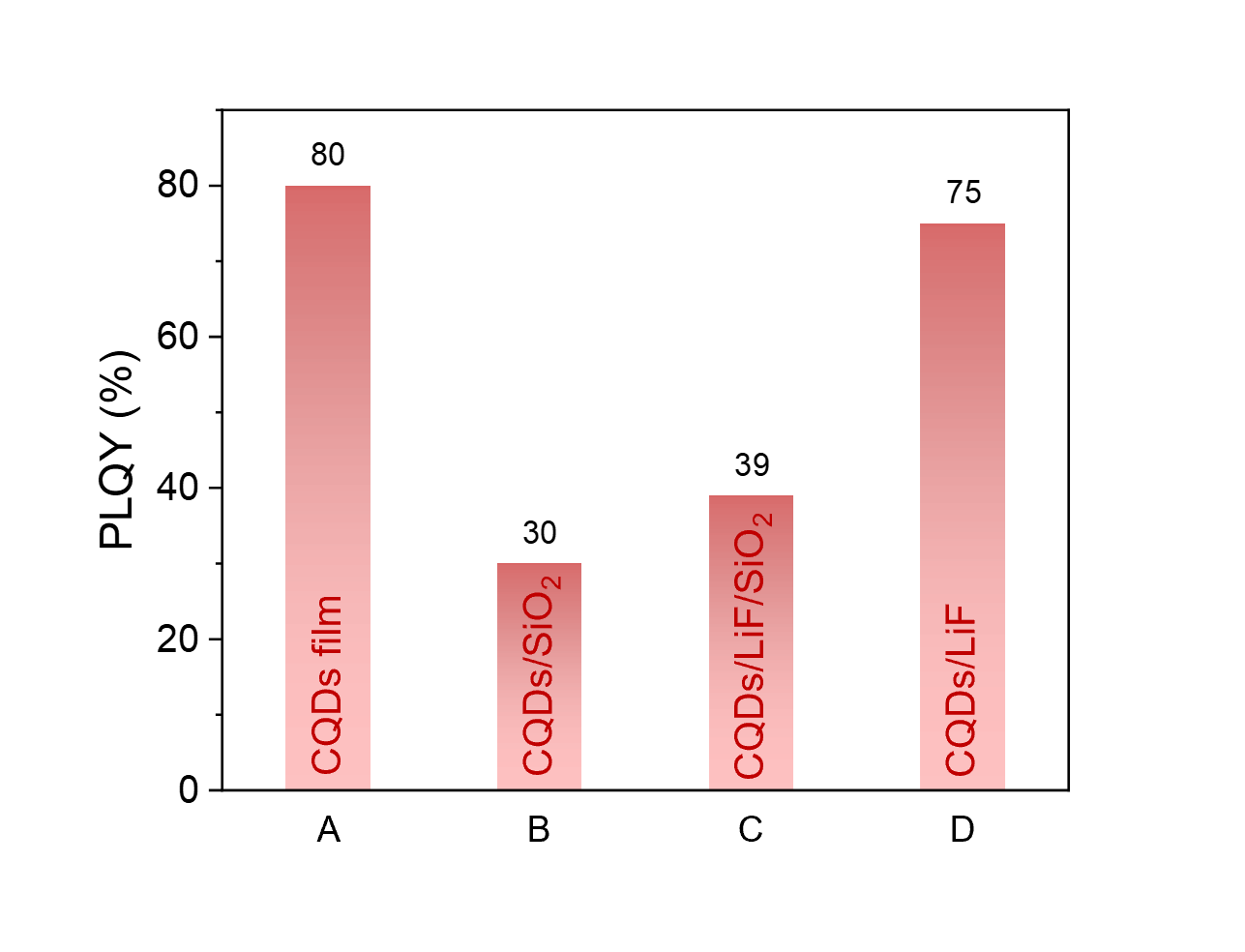


The initial PLQY for QDs on quartz substrate is 80% (sample A). After depositing SiO_2_ on QDs via magnetron sputtering, the PLQY reduced significantly to 30% (sample B). This deterioration is likely attributed to the high chamber temperature (>150°C) and high-energy particle bombardment associated with the magnetron sputtering process. Even when employing a thermal-evaporated LiF layer as a buffer between the QDs and SiO_2_, the PLQY of QDs still experienced a significant decline of over 50% (sample C).

In contrast, using only thermally evaporated LiF as the spacer layer preserved the PLQY at 75%, corresponding to 94% of the initial value (sample D). These results suggest that thermal-evaporated LiF is a more suitable choice for the spacer layer due to its minimal impact on QD emission. Additionally, thermal evaporation can also be used to deposit the top Ag thin film, simplifying the fabrication process.

Figure S11. Simulation of the optical field distribution within the cavity

**(A)** The optical field distribution of 559 nm and 630 nm in cavity without LiF.

**(B)** The optical field distribution of 559 nm and 630 nm in cavity with 60 nm LiF.

**(C)** The partial optical field distribution of 559 nm and 630 nm in cavity without LiF.

**(D)** The partial optical field distribution of 559 nm and 630 nm in cavity with 60 nm LiF.

The Γ_Ag_ and Γ_QD_ are defined as the energy ratio confined in the corresponding region to that in the entire cavity:

$$\begin{matrix} \Gamma_{QD}=\frac{\int_{QD\text{ }} \left|E(z)\right|^{2}dz}{\int_{cavity\text{ }} \left|E(z)\right|^{2}dz} \\ \Gamma_{Ag}=\frac{\int_{Ag\text{ }} \left|E(z)\right|^{2}dz}{\int_{cavity\text{ }} \left|E(z)\right|^{2}dz} \end{matrix}$$

The nonradiative SPP loss is positively related to the Γ_Ag_. The introduced 60 nm LiF spacer layer helps separate the antinode of the optical field in cavity from the lossy silver thin film, therefore reducing the Γ_Ag_ (630 nm) from 1.7% to 0.4% compared with the control sample without LiF.

On the other hand, the localization of electric field in the QDs active region would lead to stronger Purcell effect. Although the Γ_QD_ of 630 nm is decreased from 25% to 20% with introduced LiF, the field antinode is closer to the center of QDs film in the optimized sample. In contrast, the field antinode in the control sample is in the interface between QDs film and SiO_2_.


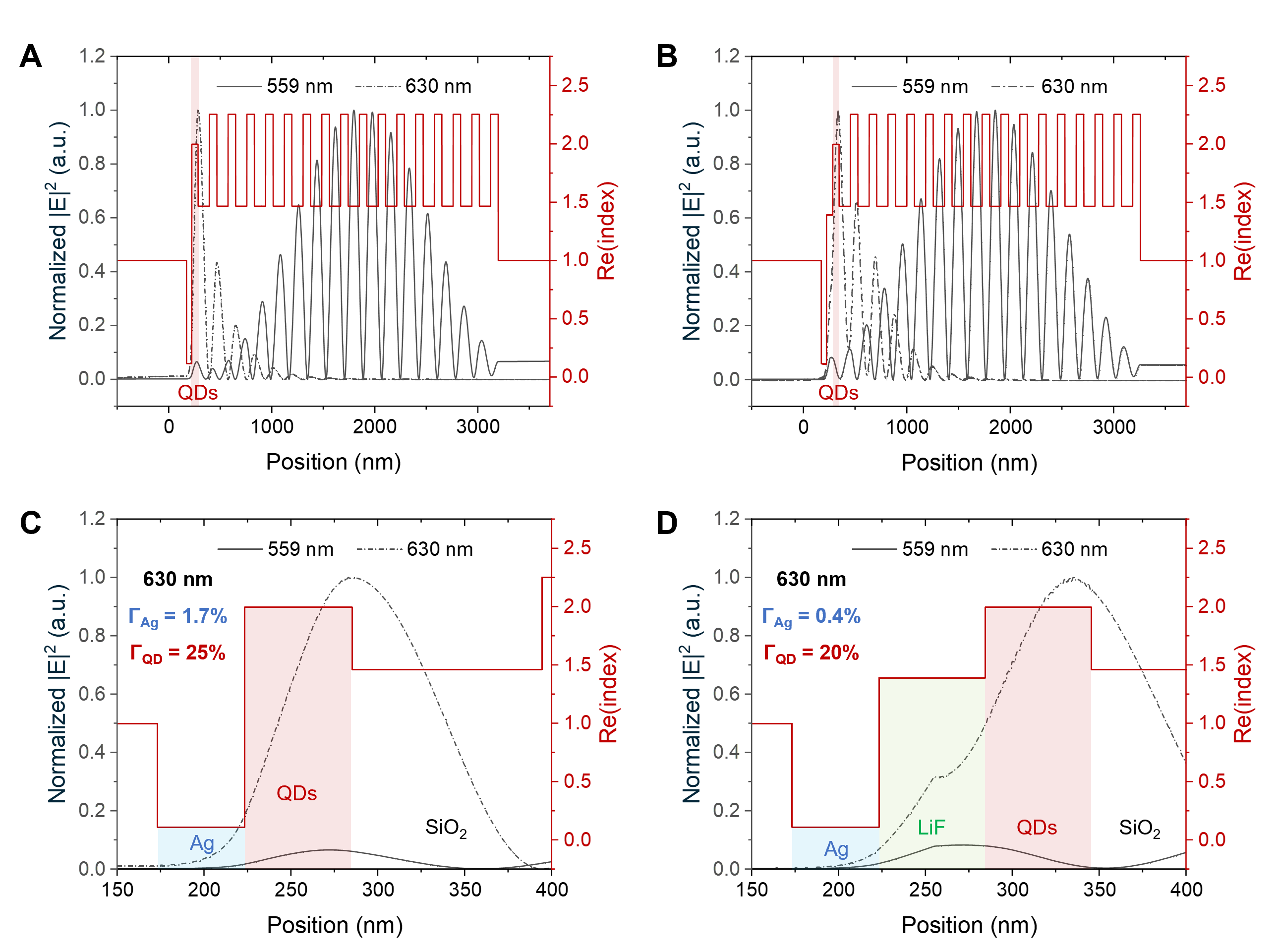


Figure S12. The spontaneous emission lifetime shorten effect in cavity

**(A)** The time-resolved PL (TRPL) decay curves of QDs film, control APE sample without LiF, optimized sample with 60 nm LiF. The signal is collected from the back (substrate) side of the sample.

**(B)** The spontaneous emission lifetime shorten factor (*k_lifetime_*) of the control and optimized samples extracted from their TRPL characteristics.


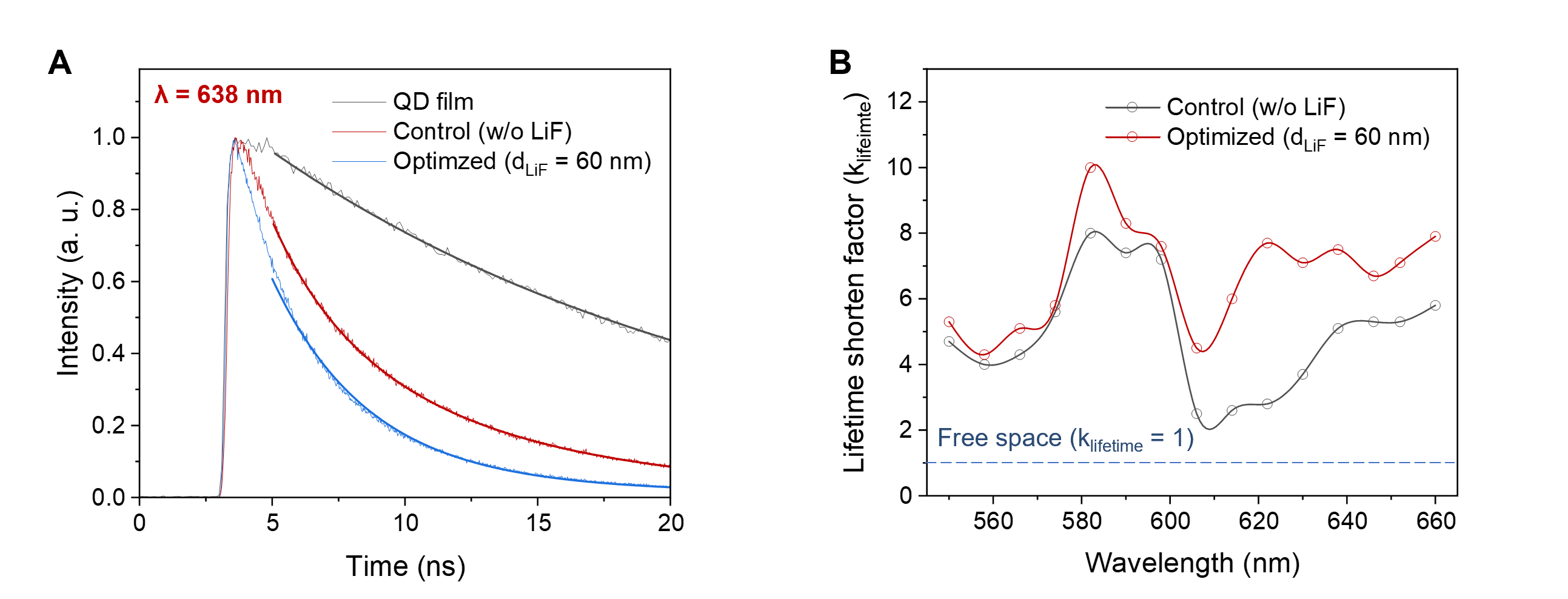


The *k_lifetime_*, also referred to as Purcell factor in some literature [3, 4], quantifies the ratio between the spontaneous emission (or, radiative recombination) rate in cavity and that in free space for the sample without LiF marked as “control” and that with 60 nm LiF marked as “optimized”. This factor can be extracted from their TRPL characteristics.

An example of the TRPL characteristics of the QDs film, control sample without LiF, optimized sample with 60 nm LiF is given in Figure S12A. The PL decay curves can be well fitted by a bi-exponential decay function:

$$I(t)=\sum_{i=1}^{2} A_{i}\exp(-t/\tau_{i})$$

where *τ_i_* is the time constant with the corresponding weight ratio *A_i_*. The two components have been ascribed to non-radiative recombination lifetime *τ_1_*, and the radiative recombination lifetime *τ_2_* [5]. Notably, the PLQY could be calculated by the ratio between the radiative recombination rate 1/*τ_2_* and the total recombination rate 1/*τ_1_*+1/*τ_2_*. For example, the PLQY of QDs film at *λ* = 606 nm is calculated to be about 74% with the fitted *τ_1_* and *τ_2_*. The *k_lifetime_*, which is defined as the ratio between spontaneous emission rate of emitter in cavity and that in free space, can be written as following in our case:

$$k_{lifetime}=\frac{1/\tau_{2,cavity}}{1/\tau_{2,film}}=\frac{\tau_{2,film}}{\tau_{2,cavity}}$$

As shown in Figure S12B, the optimized sample exhibits about twice *k_lifetime_* compared with the control sample ranging from *λ* = 606 nm to 638 nm, indicating a stronger red spontaneous emission from the optimized sample than the control one.

Figure S12. The spontaneous emission lifetime shorten effect in cavity (extended)

The fitted *τ_1_*, *τ_2_* and calculated *k_lifetime_* of the QDs film, control sample without LiF and optimized sample with 60 nm LiF at different wavelengths are listed below:


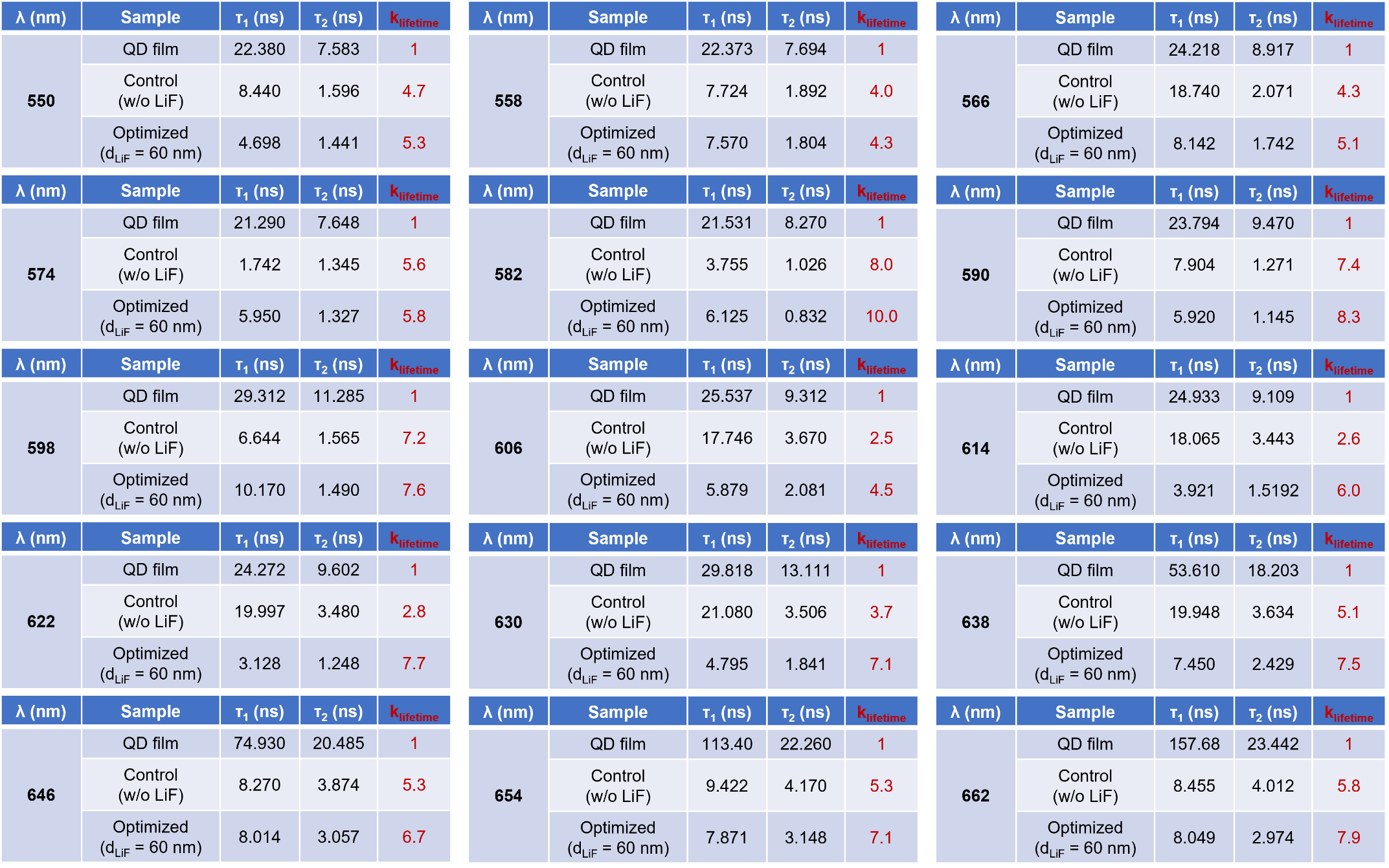


Figure S13. The pumping intensity dependent APE phenomenon of cavity coupled QDs with 60 nm LiF spacer layer (optimized sample)

**(A, B)** The emission spectrum of the cavity coupled QDs with 60 nm LiF spacer layer under pumping fluence *P* from 21 to 1236 μJ cm^-2^ at *θ* = 0.

**(C, D)** The emission spectrum of the cavity coupled QDs with 60 nm LiF spacer layer under pumping fluence *P* from 21 to 1236 μJ cm^-2^ at *θ* = 55°.


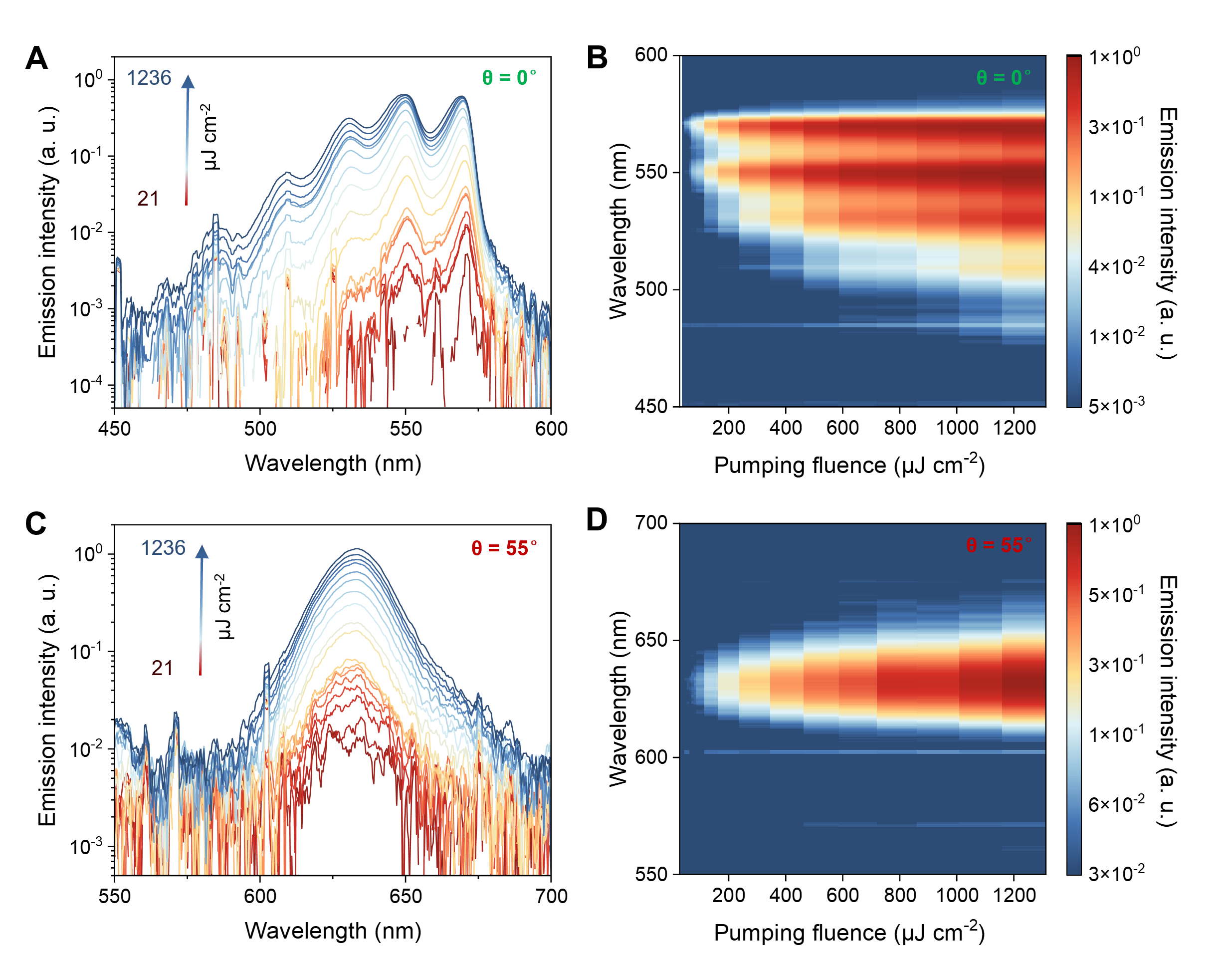


Figure S14. Repeatability and statistics of the control and optimized samples

**(A)** PL spectra of nine control samples.

**(B)** PL spectra of nine optimized samples.

**(C)** Distribution of APE threshold (*P_th,APE_*) of the control and optimized samples.

**(D)** Statistics of *P_th,APE_* of the control and optimized samples.


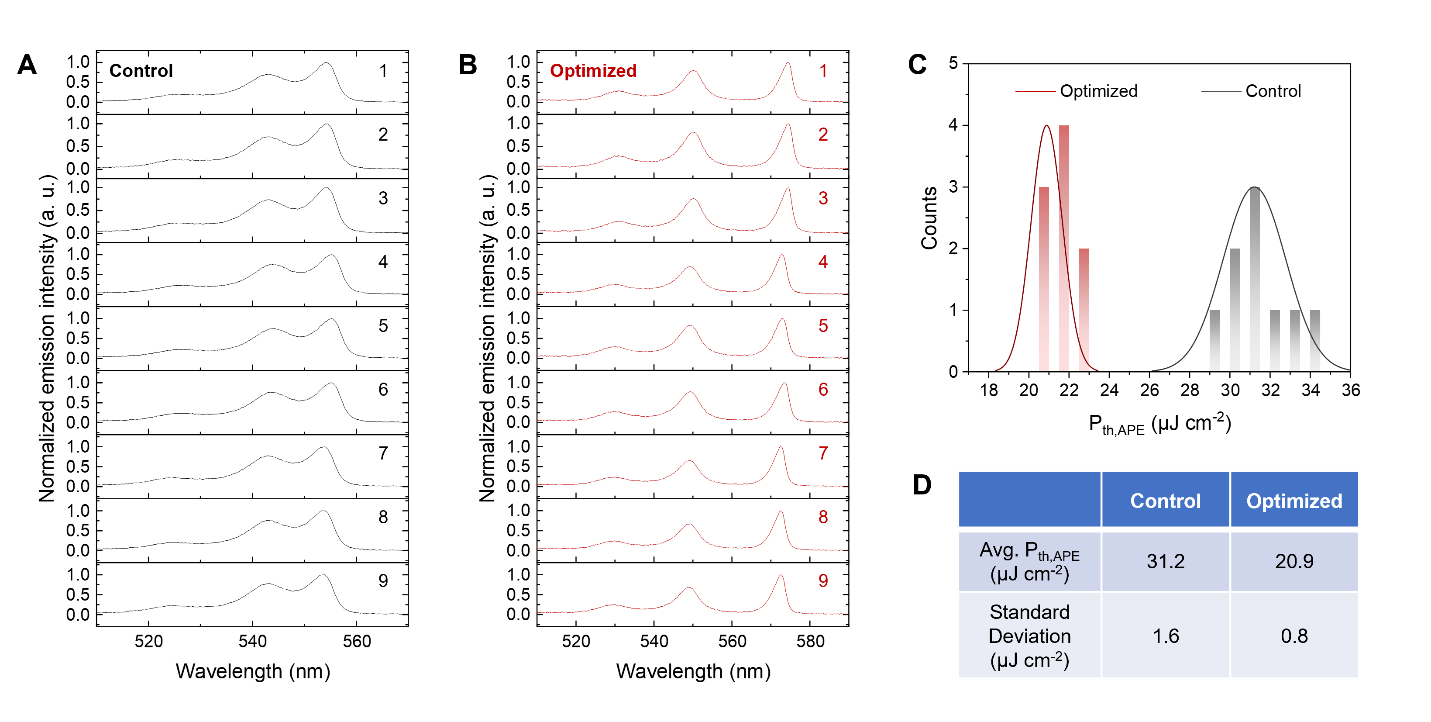


Figure S15. The color gamut coverage of the LD-pumped APE-based micro-display


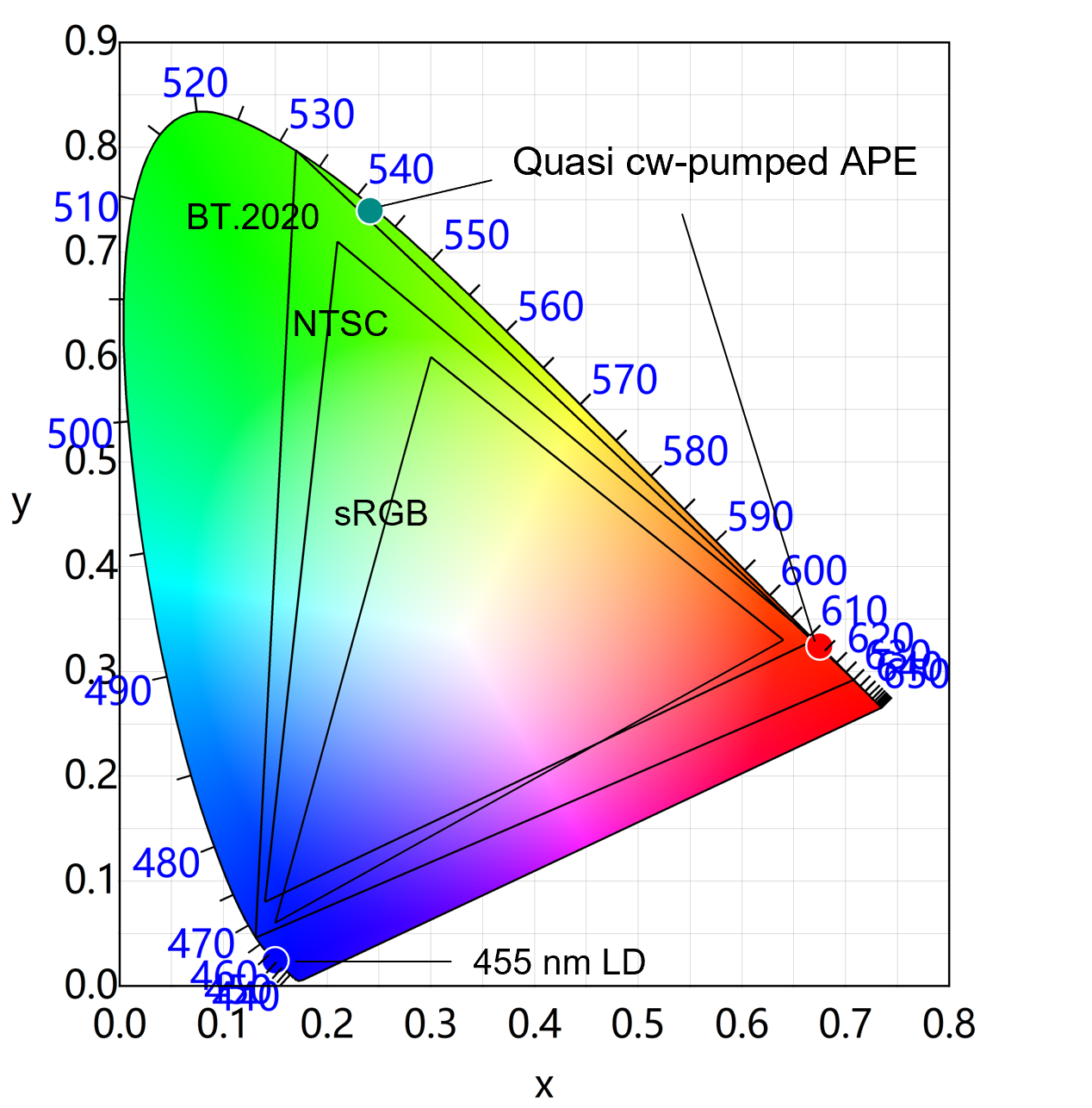


The green point at [0.242, 0.739] and red point at [0.686, 0.312] correspond to the emission of the cavity-coupled QDs and QDs film on DBRs at *θ* = 0 under *E* = 1560 W cm^-2^, respectively.

Table S1. Comparison among the reported different types of multi-excitonic polychromatic emission (PE) from monodisperse colloidal QDs


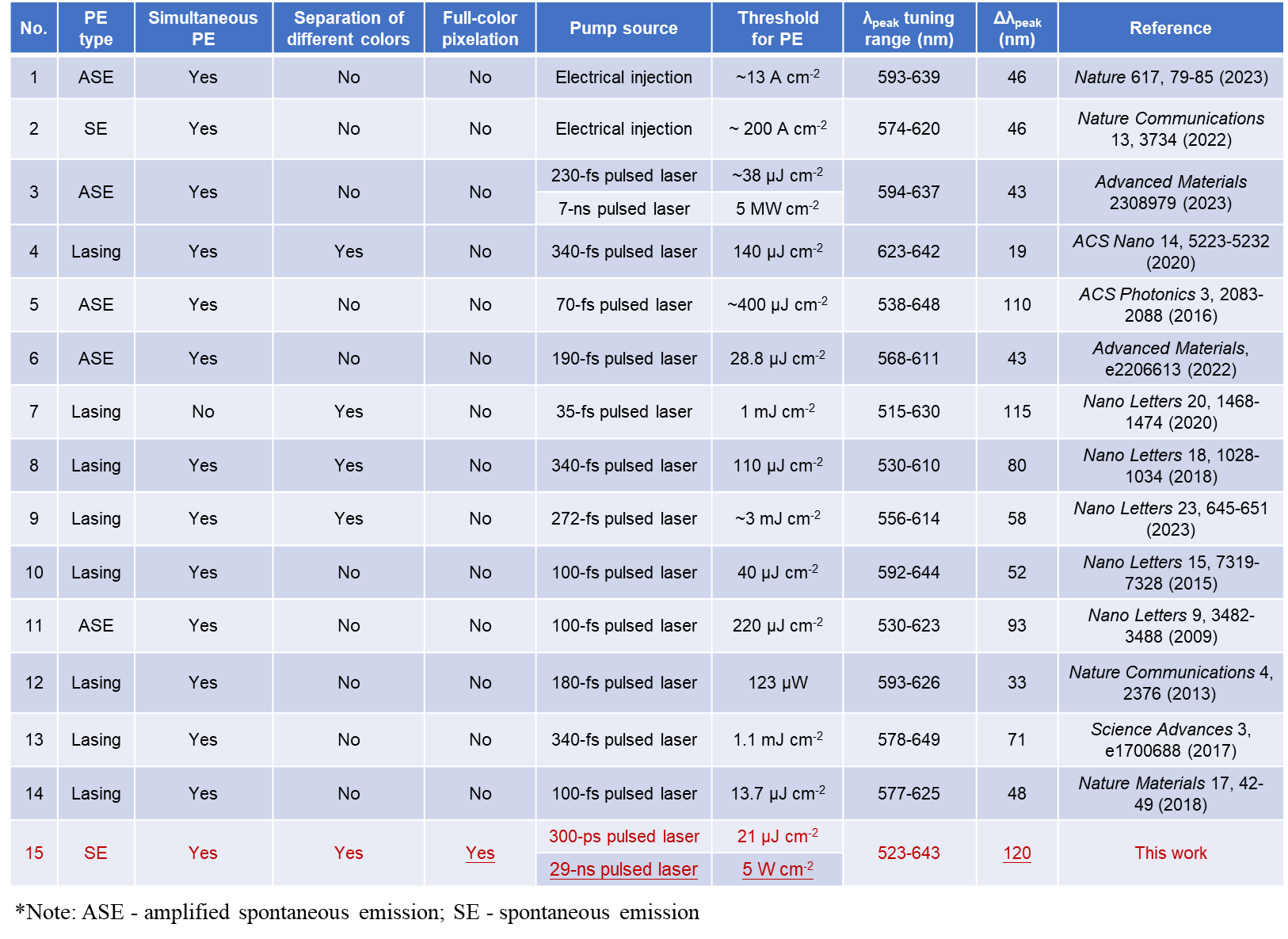


Movie S1.

Note: the APE sample is flipped to emit light from its back (substrate) side.

References

1. Klimov VI, Ivanov SA, Nanda J *et al.* Single-exciton optical gain in semiconductor nanocrystals. *Nature*. 2007; **447**(7143): 441-446. doi: 10.1038/nature05839

2. Choi AH. *Handbook of optical microcavities*: CRC Press, 2014.

3. Purkayastha P, Gallagher S, Jiang Y *et al.* Purcell Enhanced Emission and Saturable Absorption of Cavity-Coupled CsPbBr3 Quantum Dots. *ACS Photonics*. 2024; **11**(4): 1638-1644. doi: 10.1021/acsphotonics.3c01847

4. Artemyev MV, Woggon U, Wannemacher R *et al.* Light Trapped in a Photonic Dot:  Microspheres Act as a Cavity for Quantum Dot Emission. *Nano Letters*. 2001; **1**(6): 309-314. doi: 10.1021/nl015545l

5. Neogi A, Lee C-W, Everitt HO *et al.* Enhancement of spontaneous recombination rate in a quantum well by resonant surface plasmon coupling. *Physical Review B*. 2002; **66**(15). doi: 10.1103/PhysRevB.66.153305
